# GDP polyribonucleotidyltransferase domain of vesicular stomatitis virus polymerase regulates leader-promoter escape and polyadenylation-coupled termination during stop-start transcription

**DOI:** 10.1371/journal.ppat.1010287

**Published:** 2022-02-02

**Authors:** Minako Ogino, Todd J. Green, Tomoaki Ogino

**Affiliations:** 1 Department of Medical Microbiology and Immunology, College of Medicine and Life Sciences, University of Toledo, Toledo, Ohio, United States of America; 2 Department of Microbiology, School of Medicine, University of Alabama at Birmingham, Birmingham, Alabama, United States of America; Boston University, UNITED STATES

## Abstract

The unconventional mRNA capping enzyme (GDP polyribonucleotidyltransferase, PRNTase) domain of the vesicular stomatitis virus (VSV) L protein possesses a dual-functional "priming-capping loop" that governs terminal *de novo* initiation for leader RNA synthesis and capping of monocistronic mRNAs during the unique stop-start transcription cycle. Here, we investigated the roles of basic amino acid residues on a helix structure directly connected to the priming-capping loop in viral RNA synthesis and identified single point mutations that cause previously unreported defective phenotypes at different steps of stop-start transcription. Mutations of residue R1183 (R1183A and R1183K) dramatically reduced the leader RNA synthesis activity by hampering early elongation, but not terminal *de novo* initiation or productive elongation, suggesting that the mutations negatively affect escape from the leader promoter. On the other hand, mutations of residue R1178 (R1178A and R1178K) decreased the efficiency of polyadenylation-coupled termination of mRNA synthesis at the gene junctions, but not termination of leader RNA synthesis at the leader-to-*N*-gene junction, resulting in the generation of larger amounts of aberrant polycistronic mRNAs. In contrast, both the R1183 and R1178 residues are not essential for cap-forming activities. The R1183K mutation was lethal to VSV, whereas the R1178K mutation attenuated VSV and triggered the production of the polycistronic mRNAs in infected cells. These observations suggest that the PRNTase domain plays multiple roles in conducting accurate stop-start transcription beyond its known role in pre-mRNA capping.

## Author summary

Vesicular stomatitis virus (VSV), an animal rhabdovirus closely related to rabies virus, has served as a paradigm for understanding the basic molecular mechanisms of transcription and replication by rhabdoviruses (e.g., rabies) and other non-segmented negative strand (NNS) RNA viruses, such as measles and Ebola. NNS RNA viral polymerases sequentially synthesize the non-coding leader RNA and monocistronic mRNAs from the 3′-terminal leader region and internal genes, respectively, on their genomes by the stop-start transcription mechanism. A hallmark of NNS RNA viral polymerases is the presence of a unique enzymatic domain, called GDP polyribonucleotidyltransferase (PRNTase), which catalyzes pre-mRNA 5′-capping, one of the essential mRNA modifications. Our recent study revealed that the VSV PRNTase domain directs transcription initiation at the 3′-end of the genome as well as pre-mRNA capping with the dual functional priming-capping loop during stop-start transcription. Here, we further show that a helix structure flanked by the priming-capping loop regulates not only transcription elongation at an early phase of leader RNA synthesis but also polyadenylation-coupled transcription termination at gene junctions. These findings indicate that the PRNTase domain acts as a key regulatory domain for stop-start transcription as well as a catalytic domain for pre-mRNA capping.

## Introduction

Vesicular stomatitis virus (VSV), a non-segmented negative strand (NNS) RNA virus belonging to the *Rhabdoviridae* family in the order *Mononegavirales*, has been used as a prototypic virus to study the molecular mechanisms of transcription and replication in this order of viruses (Reviewed in [1]). During transcription, the VSV RNA-dependent RNA polymerase (RdRp) complex comprising the large (L) protein and its co-factor phospho- (P) protein enters from the 3′-end of the genomic RNA template encapsidated with the nucleocapsid (N) protein (called N-RNA template) to synthesize the uncapped leader RNA (LeRNA) with ~47 nucleotides (nt) [2–5]. After synthesis of LeRNA, the RdRp sequentially transcribes five internal genes (*N*, *P*, *M*, *G*, and *L*) into monocistronic mRNAs with a 5′-cap 1 structure [m^7^G(5′)ppp(5′)Am-, m^7^G, *N*^7^-methylguanosine; Am, 2′-*O*-methyladenosine] and 3′-poly(A) tail via a stop-start transcription mechanism [4,6–8]. The internal genes with the conserved gene-start (3′-UUGUCDNUAG; D: A, U, or G; N: any nucleotide) and gene-end (3′-AUACUUUUUUU) sequences are tandemly linked via 2-nt intergenic regions (3′-SA; S: G or C). The gene-start and gene-end sequences serve as transcription initiation and polyadenylation/termination signals, respectively [9,10]. Since transcription reinitiation at each gene junction is ~70% [11], stop-start transcription generates a gradient in mRNA abundance in the following order: *N* > *P* > *M* > *G* > *L* [7,8]. In contrast, during replication, these signals for mRNA synthesis on the genome are ignored by the RdRp to synthesize a full-length anti-genome, which is known to be co-replicationally encapsidated with the N proteins [12–15].

In rhabdoviruses, such as VSV [16,17], Chandipura virus [18], and rabies virus (RABV) [19], the formation of the cap core structure [G(5′)ppp(5′)A-] proceeds via the stepwise actions of the guanosine 5′-triphosphatase (GTPase) and GDP polyribonucleotidyltransferase (PRNTase, EC. 2.7.7.88) activities of their L proteins. In the first step, the GTPase activity associated with the L protein removes the γ-phosphate of GTP to produce GDP, which in turn serves as a 5′-monophospho-RNA (pRNA) acceptor substrate for the subsequent PRNTase reaction [16,20]. In the second step, the PRNTase domain in the VSV L protein specifically recognizes a 5′-triphosphorylated pre-mRNA (pppRNA) with the conserved mRNA start-sequence (5′-AACAG-) complementary to the gene-start sequence and then transfers its pRNA moiety to GDP via a covalent enzyme–pRNA (called L–pRNA) intermediate to produce a capped pre-mRNA (GpppRNA) [16,17].

The PRNTase domain (residues 1081–1331) of the VSV L protein (2109 amino acids) possesses five collinear motifs, Rx(3)Wx(3–8)ΦxGxζx(P/A) (motif A), (Y/W)ΦGSxT (motif B), W (motif C), HR (motif D), and ζxxΦx(F/Y)QxxΦ (motif E) (Φ, hydrophobic; ζ, hydrophilic amino acids), which are conserved in NNS RNA viral L proteins [1,21]. In the atomic structure of the VSV L protein (PDB id: 5A22) solved by cryo-electron microscopy (EM) [22], these motifs are located in close proximity to build an active site of the PRNTase domain [21]. We identified residues G1100 (motif A), T1157 (motif B), W1188 (motif C), H1227 (motif D), R1228 (motif D), F1269 (motif E), and Q1270 (motif E) in the VSV PRNTase domain as important or essential for the capping reaction at the step of the L–pRNA intermediate formation [17,21]. All these key residues are critical for VSV gene expression and propagation in host cells [21,23]. H1227 in motif D serves as a catalytic amino acid residue, in which the *N*^ε2^ position of the imidazole ring is covalently linked to the 5′-terminal α**-**phosphorus atom of pRNA with a phosphoamide bond during the intermediate formation [17]. The pRNA linked to H1227 is specifically transferred to GDP, resulting in the release of GpppRNA from the PRNTase domain [17,24].

In our reconstituted transcription system, cap-defective mutations of the key residues in the PRNTase motifs have no or modest effects on LeRNA synthesis, but trigger termination of *N* mRNA synthesis at fixed positions +38 and +40 of the *N* gene to generate 5′-triphosphorylated short transcripts (called N1–38 and N1–40, respectively) [21,23]. After synthesis of N1–38 or N1–40, these cap-defective L mutants conduct aberrant stop-start transcription using cryptic transcription initiation and termination signals within the *N* gene to generate unusual 5′-pppG-initiated transcripts, such as N41–68 and 3′-polyadenylated N157–1326 [23]. These observations suggest that successful pre-mRNA capping at an early stage of mRNA chain elongation is required to prevent termination of mRNA synthesis at position +38 or +40 leading to aberrant stop-start transcription. Similarly, mutations of PRNTase motif B or D in the L protein of human respiratory syncytial virus (HRSV, *Pneumoviridae*) were reported to cause premature termination of mRNA synthesis to generate ~40-nt abortive transcripts [25].

The PRNTase domain in the apo-structure (i.e., in absence of RNA) of the VSV L protein possesses a large loop structure that is deeply inserted into an active site cavity of the RdRp domain on the same polypeptide [22]. By analogy with primer-independent RdRps of other unrelated RNA viruses [26–28], the loop of the VSV PRNTase domain was suggested to serve as a priming loop, which might play a critical role in transcription initiation [22]. We biochemically demonstrated that the loop of the VSV PRNTase domain governs not only terminal *de novo* initiation to synthesize LeRNA but also pre-mRNA capping [29]. Thus, the loop of the VSV PRNTase domain acts as a dual-functional “priming-capping loop”. We identified W1167 on the priming-capping loop of the VSV PRNTase domain as essential for terminal *de novo* initiation, but not for pre-mRNA capping [29]. The RABV L counterpart (W1180) of W1167 is critical for terminal *de novo* initiation, but not for internal *de novo* initiation from the RABV gene-start sequence or RNA chain elongation [29]. Interestingly, although the TxΨ motif (VSV, T1161-x-I1163; RABV, T1174-x-L1176) on the loop is far from the PRNTase active site in the apo-structures of rhabdoviral L proteins [22,30], this motif is essential for capping in the step of the intermediate formation, but not for transcription initiation [29]. These findings suggest that the priming-capping loop undergoes a structural rearrangement to mediate pre-mRNA capping during stop-start transcription.

Recent cryo-EM analyses of L proteins (complexed with their cognate P proteins) of HRSV [31], human metapneumovirus (HMPV, *Pneumoviridae*) [32], and parainfluenza virus 5 (PIV5, *Paramyxoviridae*) [33] belonging to the order *Mononegavirales* revealed that their putative PRNTase domains harbor their counterparts of the rhabdoviral priming-capping loop. However, their loop structures are arranged in distinct configurations and are associated with or close to their putative PRNTase active sites [31–33]. These observations further support our prediction that the rhabdoviral priming-capping loop is flexible during stop-start transcription and can change its configuration into a form that mediates the L–pRNA intermediate formation in the pre-mRNA capping reaction [29].

Although accumulating evidence suggests that the PRNTase domain is required for multiple steps of viral RNA synthesis, its precise roles in transcription and replication have not been fully explored. Here, we performed site-directed mutagenesis to analyze functions of basic amino acid residues close to the VSV PRNTase active site in RNA biosynthesis, and identified two amino acid residues that regulate stop-start transcription at previously unappreciated steps. We propose new functions of the PRNTase domain that are independent of its known role in pre-mRNA capping.

## Results

### The priming-capping loop in the PRNTase domain is anchored to a highly basic helix structure

**Fig 1:**
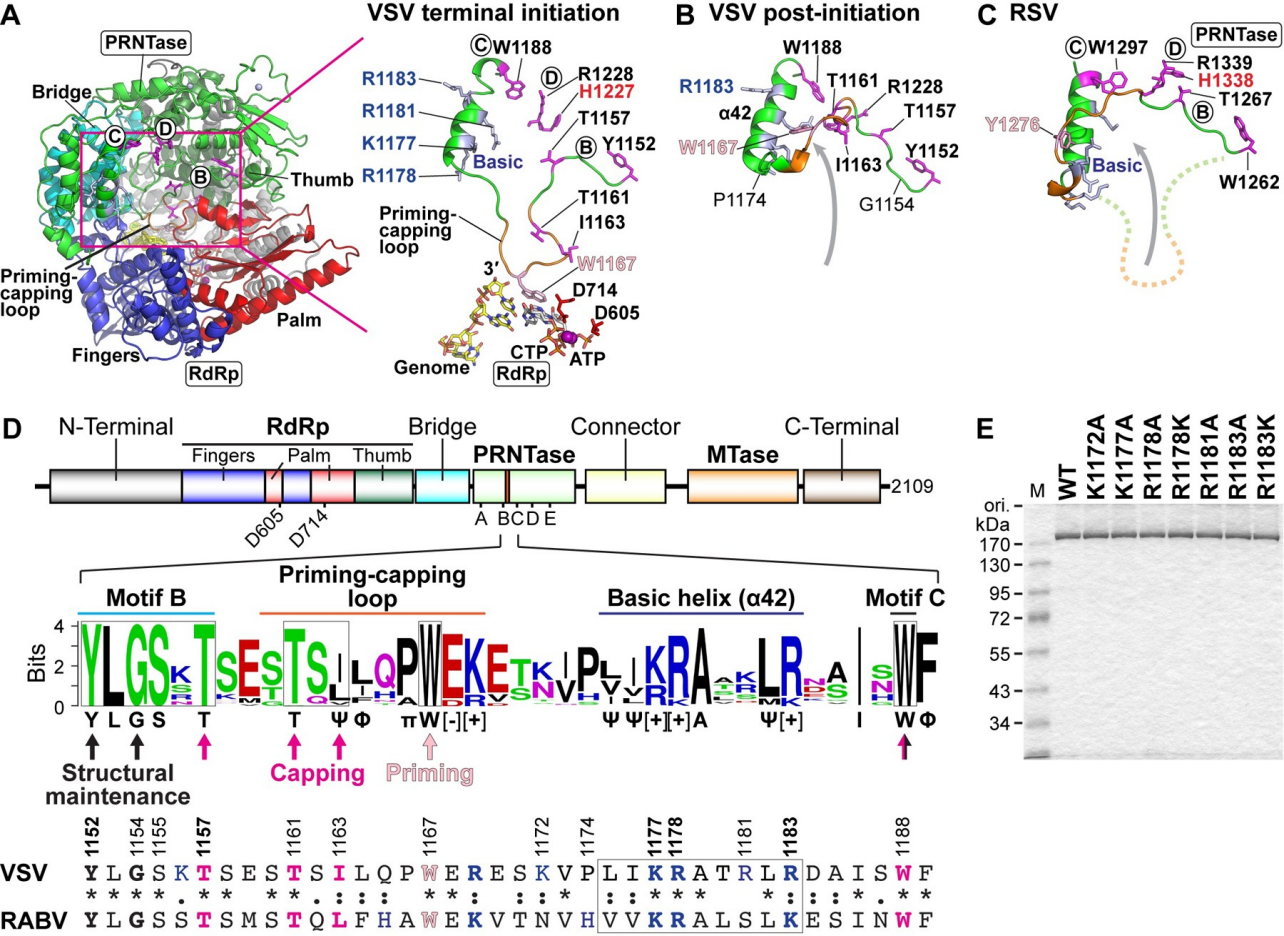
The priming-capping loop is anchored to a highly basic α-helix structure in the VSV PRNTase domain. (**A**) A structural model of the RdRp (fingers subdomain, blue; palm subdomain, red; thumb subdomain, dark green) and PRNTase (green) domains of the VSV L protein in a terminal initiation complex (Model Archive id: ma-5k432) is shown as a ribbon diagram (left). Key amino acid residues (T1161, I1163, W1167) on the priming-capping loop (orange) and flanking helix, active site amino acid residues in the RdRp (D605, D714) and PRNTase (Y1152 and T1157 in motif B, W1188 in motif C, H1227 and R1228 in motif D) domains, the 3′-terminal of the genome (3′-UGCU-5′), and initiator (ATP) and incoming (CTP) nucleotides are shown in stick model (close-up view, right). (**B** and **C**) A putative post-initiation conformation of the VSV priming-capping loop (**B**) was modeled based on the structure of its HRSV counterpart (**C**) in the apo-state of the HRSV L-P RdRp complex (PDB id: 6PZK). For context in (**C**), the priming-capping loop in a putative initiation state conformation is shown in a dashed-line. (**D**) A schematic structure of the VSV L protein is shown with proposed domains/subdomain [1] (upper). MTase indicates methyltransferase. The positions of RdRp catalytic residues (D605 and D714) and PRNTase motifs (A–E) are indicated. An amino acid sequence logo for regions ranging from PRNTase motif B to motif C in L proteins of vertebrate and arthropod rhabdoviruses (excluding novirhabdoviruses) was generated by the WebLogo program (middle). Conserved amino acid residues are shown below the logo. Ψ, Φ, π, [–], and [+] indicate aliphatic, hydrophobic, small, acidic, and basic amino acid residues, respectively. Amino acid residues required for the mRNA capping activity, terminal *de novo* initiation (priming) activity, and structural maintenance of the VSV PRNTase domain are marked by magenta, pink, and black arrows, respectively [21,29]. Local amino acid sequences of the VSV and RABV L protein are shown (lower). The numbers indicate positions of the amino acid residues of the VSV L protein. (**E**) Purified recombinant VSV L proteins with a point-mutation in the indicated basic amino acid residues (1 μg) were analyzed alongside with the wild-type (WT) L protein by 7.5% SDS-PAGE followed by staining with One-Step Blue. The names of the point-mutants include the original amino acid (one-letter code) at the indicated position in the VSV L protein followed by the replacement amino acid. M lane shows molecular size marker.

We have previously modeled a structure of a VSV L terminal initiation complex with a model RNA (the 3′-terminal sequence of the VSV genome) and initiator and incoming nucleotides (ATP and CTP, respectively) based on the structures of the VSV L in the apo-state and bacteriophage Φ6 RdRp initiation complex [29] (**Fig 1A**). In the structural model, the adenine ring of ATP is sandwiched between the indole ring of W1167 and the cytosine ring of CTP via π- π stacking interactions to stabilize the terminal initiation complex formed on the 3′-terminal UG sequence of the template RNA. Here, we modeled a structure of the priming-capping loop in a putative post-initiation state (**Fig 1B**) based on the structure of the PRNTase-like domain of the HRSV L protein complexed with the P protein in the apo-state (**Fig 1C**) [31]. The N-terminal end of the rhabdoviral priming-capping loop is flanked by PRNTase motif B, in which Y1152 interacts with Q1270 in motif E via hydrogen bonding and G1154 serves as a structural transition point to begin the loop, allowing it to orient into the catalytic channel. On the other hand, the C-terminal end of the priming-capping loop transitions out of the cavity to anchor to an α-helix structure (α42, residues 1175–1186), which is flanked by motif C and is closely juxtaposed to the catalytic HR motif (motif D). In the putative post-initiation state (**Fig 1B**), the modeled priming-capping loop is pivoted up and out of the channel at the point of G1154/S1155 to sit along the PRNTase active site and the upper rim of the putative exit channel for newly formed RNA. Thus, G1154 could be considered a pivot point for motion of the priming-capping loop on the N-terminal end. On the C-terminal end, P1174 is heavily rotated between the initiation and post-initiation states, serving as a transition point into the rigid secondary structure of helix α42. In the post-initiation state, the priming-capping loop makes contact with the helix structure α42. One potential interaction for stabilizing this loop conformation, as suggested by the model, is the hydrogen bonding network generated between loop residue E1159 with R1181 and S1235. Helix α42 is heavily charged, presenting a series of basic amino acid residues K1177, R1178, R1181, and R1183.

We analyzed local amino acid sequences between PRNTase motif B and motif C in L proteins of 109 vertebrate and arthropod rhabdoviruses (**Fig 1D**). As we reported [29], this region includes the priming-capping loop with the conserved TxΨ motif (for VSV, T1161-x-I1163) and tryptophan (W1167) residue. We found that the helix structure between the priming-capping loop and motif C is mainly composed of aliphatic and basic amino acid residues with conservative substitutions. Since the basic helix not only forms a putative GDP binding cavity for the capping reaction [1] but also serves as an anchoring structure for the flexible priming-capping loop, we explored the roles of the basic amino acid residues (K1177, R1178, and R1183) on the helix in RNA capping and synthesis. For this purpose, we generated recombinant VSV L proteins with a single-point mutation in these basic residues and flanking non-conserved basic amino acid residues (K1172 and R1181) using a baculovirus expression system (**Fig 1E**).

### Basic amino acid residues on the priming-capping loop-anchoring helix have regulatory, but not catalytic, functions in mRNA capping

**Fig 2:**
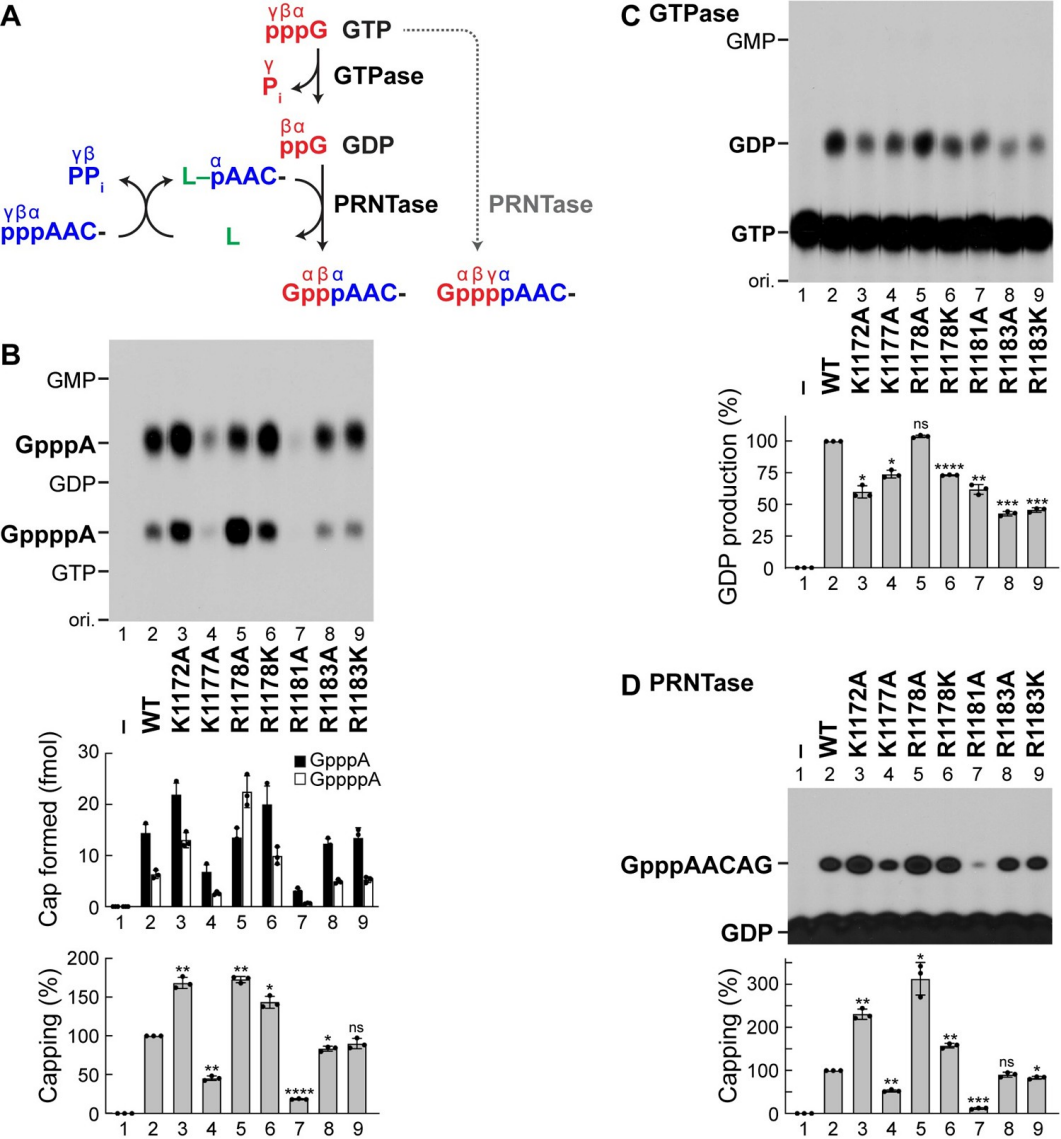
Mutations of the semiconserved basic amino acid residues in the α-helix structure do not abolish the cap-forming activities of the VSV L protein. (**A**) The unconventional pathway of the VSV mRNA cap formation is schematically depicted. The guanosine 5′-triphosphatase (GTPase) activity associated with the VSV L protein hydrolyzes GTP (pppG) into GDP (ppG). The GDP polyribonucleotidyltransferase (PRNTase) domain of the VSV L protein transfers 5′-monophosphorylated RNA (pAAC-) from 5′-triphosphorylated RNA (pppAAC-) to GDP to generate the cap core structure G(5′)ppp(5′)A- (center product). When pAAC- is transferred to GTP, G(5′)pppp(5′)A- is formed (right product). GTP and RNA are shown in red and blue, respectively. The positions of 5′-phosphate groups are labeled by α, β, and γ. P_i_ and PP_i_ indicate inorganic phosphate and pyrophosphate, respectively. L denotes the L protein. (**B**) The *in vitro* capping assay was performed with recombinant VSV L protein (WT or mutant), [α-^32^P]GTP, and pppAACAG oligo RNA. Alkaline phosphatase- and nuclease P1-resistant products were analyzed by PEI-cellulose TLC followed by autoradiography (top). Lane 1 indicates no L. The positions of the origin (ori.), GpppA, GppppA, GMP, GDP, and GTP are shown. Amounts of GpppA and GppppA (closed and open columns, respectively) formed on RNA are indicated (middle). Relative cap (GpppA + GppppA) formation activities of the mutants are expressed as percentages of the activity of the WT L protein (bottom). The dot-plots, columns, and error bars represent the individual values, means, and standard deviations, respectively (n = 3). Statistical significance was determined by one-way ANOVA [ns, not significant (p ≥ 0.05); *, p < 0.05; **, p <0.01; ***, p < 0.001; ****, p < 0.0001; compared to control (WT)]. (**C**) The *in vitro* GTPase assay was carried out with recombinant VSV L protein (WT or mutant) and [α-^32^P]GTP. The reaction mixtures were analyzed by PEI-cellulose TLC followed by autoradiography (top). Relative GTPase activities of the mutants are expressed as percentages of the activity of the WT L protein (bottom). (**D**) The *in vitro* capping assay was performed using [α-^32^P]GDP, instead of [α-^32^P]GTP, as a pRNA acceptor substrate to measure PRNTase activities of the WT and mutant L proteins independently of their GTPase activities. Capped RNA products were analyzed by 20% urea-PAGE followed by autoradiography (top). Relative cap (GpppA) formation activities of the mutants are expressed as percentages of the activity of the WT L protein (bottom).

First, we examined the effects of mutations of these basic amino acid residues on oligo-RNA capping using [α-^32^P]GTP as a substrate. To generate the GpppA cap structure on the 5′-end of the AACAG oligo RNA, both the GTPase and PRNTase activities of the L protein are necessary [16,20] (**Fig 2A**). Under the *in vitro* conditions optimized for the PRNTase activity rather than the GTPase activity of the L protein [20], the wild-type (WT) L protein is known to produce GpppA and GppppA as major and minor products, respectively, as shown in **Fig 2B** (lane 2). The latter product can be generated by the direct transfer of pRNA to GTP prior to hydrolysis of GTP into GDP [20]. Interestingly, the K1172A (lane 3), R1178A (lane 5), and R1178K (lane 6) mutations increased synthesis of total cap structures (GpppA plus GppppA) to approximately 170, 170, and 140% of the WT level, respectively, but only the R1178A mutant produced a larger amount of GppppA than GpppA (lane 5). On the other hand, the K1177A (lane 4) and R1181A (lane 7) mutations reduced the capping activity, whereas the R1183A (lane 8) and R1183K (lane 9) mutations had little effect on the activity.

To analyze which step(s) of the capping reaction was affected by these mutations, we performed the GTPase and PRNTase assays separately. The GTPase reaction was monitored using [α-^32^P]GTP as a substrate (**Fig 2C**). Although we initially investigated whether the R1178A mutation impairs the production of GDP from GTP, the GTPase activity of the R1178A mutant (**Fig 2C**, lane 5) was almost the same as that of the WT enzyme (lane 2). Therefore, it seems likely that the R1178A mutant utilizes GTP as a pRNA acceptor more efficiently than the WT L protein. On the other hand, the other mutants (lanes 3, 4, 6–9) showed moderately lower GTPase activities than that of the WT L protein (lane 2). To measure PRNTase activities of the mutants independently of their GTPase activities, we performed the oligo-RNA capping assay using [α-^32^P]GDP as a pRNA acceptor substrate (**Fig 2D**). Relative activities of these mutants to produce GpppA with GDP (**Fig 2D**, lanes 3–9) were roughly consistent with their total cap synthesis activities with GTP (**Fig 2B**, lanes 3–9). The K1172A (**Fig 2D**, lane 3), R1178A (lane 5), and R1178K (lane 6) mutations enhanced the GpppA formation with GDP to approximately 230, 310, and 160% of the WT level, respectively. It was also confirmed that the R1181A mutant retains approximately 12% of the WT activity (**Fig 2D**, lane 7). Although some mutations of these basic amino acid residues in the helix structure were found to modulate the cap synthesis activities, none of these residues were critical for both the GTPase and PRNTase reactions.

### Basic amino acid residues in the priming-capping loop-anchoring helix regulate unique steps of stop-start transcription

**Fig 3:**
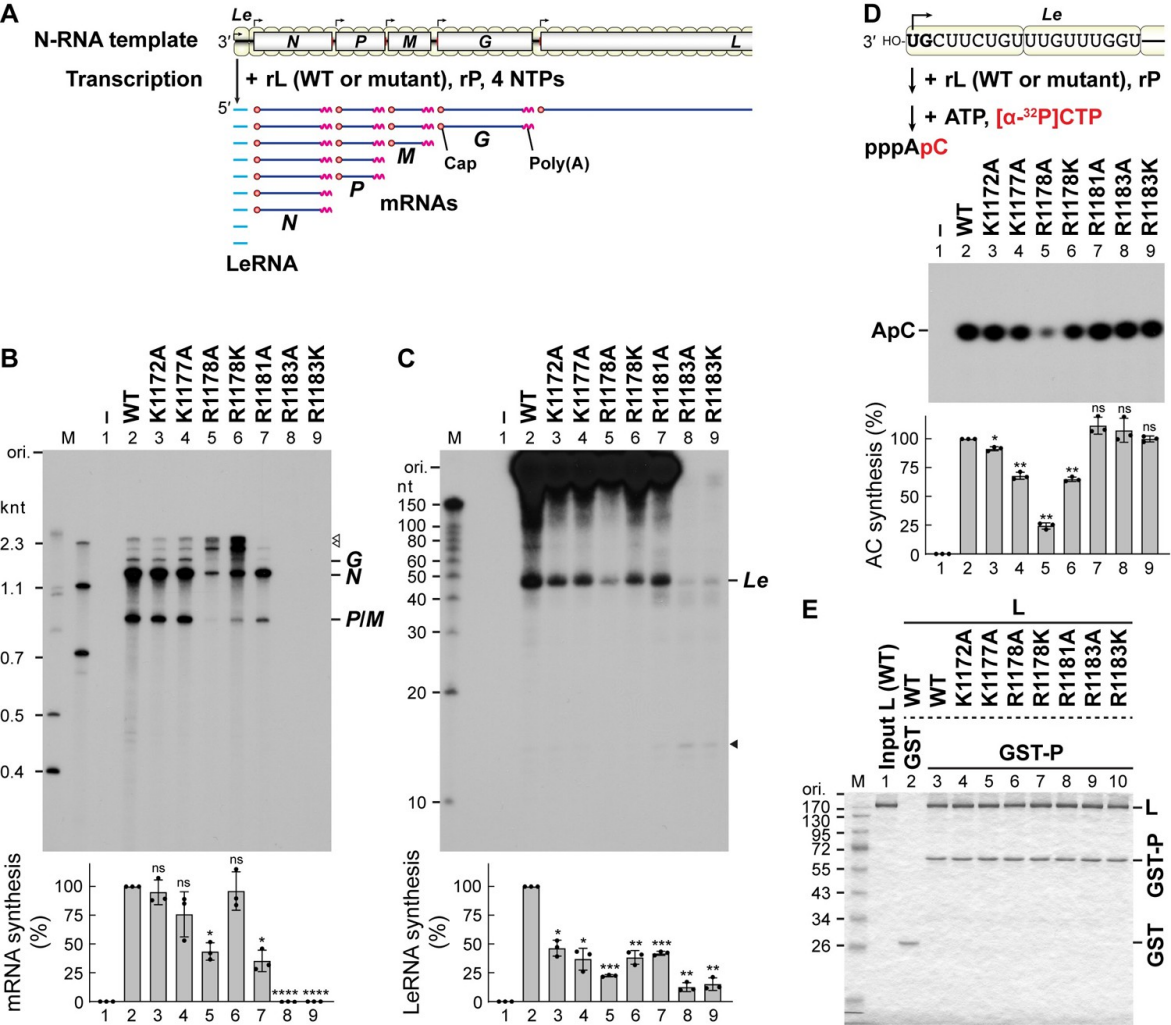
The R1178 and R1183 mutations in the VSV L protein impact distinct steps of stop-start transcription without affecting P-binding. (**A**) *In vitro* transcription was carried out with the N-RNA template (upper) and recombinant L (WT or mutant) and P proteins. The reconstituted L-P RdRp complex enters from the 3′-end of the genomic RNA and sequentially synthesizes leader RNA (LeRNA) and mRNAs with a 5′-cap structure and 3′-poly(A) tail from the 3′-leader region and internal genes, respectively (lower). Transcription initiation and polyadenylation/termination signals (gene-start and gene-end sequences, respectively) on the genome are shown by bent arrows and red vertical lines, respectively. The positions of negative-strand open reading frames are indicated by boxes. (**B** and **C**) ^32^P-labeled mRNAs and LeRNA synthesized by the WT or mutant L protein were analyzed by 5% (**B**) and 20% (**C**) urea-PAGE, respectively, followed by autoradiography (top). Lane 1 indicates no L. The positions of *N*, *P*, *M*, and *G* mRNAs and LeRNA (*Le*) are indicated on the right. Note that *P* and *M* mRNAs co-migrated at the same position (*P*/*M*). The positions of unknown long transcripts and a prematurely-terminated short transcript are marked by open (**B**) and closed (**C**) arrowheads, respectively. M lanes show marker RNAs with the indicated lengths. Relative mRNA and LeRNA synthesis activities of the mutants are expressed as percentages of the activity of the WT L protein (bottom). The dot-plots, columns, and error bars represent the individual values, means, and standard deviations, respectively (n = 3). Statistical significance was determined as in Fig 2B. (**D**) *In vitro* AC dinucleotide synthesis was performed with the recombinant L (WT or mutant) and P proteins and the N-RNA template (top). CIAP-resistant products were analyzed by 20% urea-PAGE followed by autoradiography (middle). Relative AC synthesis activities are shown (bottom). (**E**) The recombinant L protein (WT or mutant) was incubated with glutathione *S*-transferase (GST) or GST-fused P protein (GST-P) immobilized on beads. Bead-bound fractions were analyzed along with input L (1 μg, lane 1) by 10% SDS-PAGE followed by staining with One-Step Blue.

In order to investigate effects of the mutations of the basic amino acid residues on transcription, we used our transcription system reconstituted with the N-RNA template and recombinant L and P proteins (**Fig 3A**). As reported [34], the WT L protein synthesized *N* [1.3 kilo nucleotides (knt)], *P* (0.8 knt), *M* (0.8 knt), and *G* (1.6 knt) mRNAs and some minor transcripts [2.1 and ~3 knt, marked by open arrowheads] (**Fig 3B**, lane 2). It should be noted that monocistronic *P* and *M* mRNAs co-migrated in the denaturing gel (labeled by *P*/*M*). The K1172A (lane 3), K1177A (lane 4), and R1181A (lane 7) mutations had small or moderate effects on mRNA synthesis, whereas the R1183A (lane 8) and R1183K (lane 9) mutations almost completely abolished mRNA synthesis. Unexpectedly, the R1178A (lane 5) and R1178K (lane 6) mutations induced increased production of unknown transcripts (e.g., 2.1- and ~3-knt RNAs) significantly longer than the monocistronic mRNAs produced by the WT L protein. These mutations reduced their LeRNA synthesis activities mildly (**Fig 3C**, lanes 3, 4, 6, and 7) or severely (lanes 5, 8, and 9). The R1183A (lane 8) and R1183K (lane 9) mutants produced smaller amounts of full-length LeRNA, while making larger amounts of a short RNA (~12 nt, marked by a solid arrowhead), compared to the WT L protein (**S1A and S1B Fig**). This suggests that the R1183A and R1183K mutant L proteins tend to prematurely terminate LeRNA synthesis during chain elongation more frequently than the WT L protein. In contrast, the R1178A mutant did not produce detectable amounts of such short RNAs (lane 5), suggesting that this mutation may affect a very early stage of LeRNA synthesis. Consistent with these observations, *de novo* initiation of LeRNA synthesis (first-phosphodiester bond formation) at the 3′-terminal of the genomic RNA was significantly diminished by the R1178A mutation (**Fig 3D**, lane 5). Thus, this R1178A mutant has multiple abnormalities in RNA synthesis and capping. In contrast, the R1178K mutant retained about 70% of the WT initiation activity (lane 6), suggesting that a basic amino acid residue at this position is required for efficient transcription initiation as well as GpppA-cap formation. On the other hand, the R1183A (lane 8) and R1183K (lane 9) mutants exhibited transcription initiation activities comparable to that of the WT L protein. In addition, all these mutant L proteins were able to interact with the essential RdRp co-factor P protein (**Fig 3E**, lanes 4–10) as in the case of the WT L protein (lane 3), suggesting that these mutations do not perturb the folding of the L protein or disrupt this essential interaction.

### The R1183 residue is required for *Le* promoter escape

**Fig 4:**
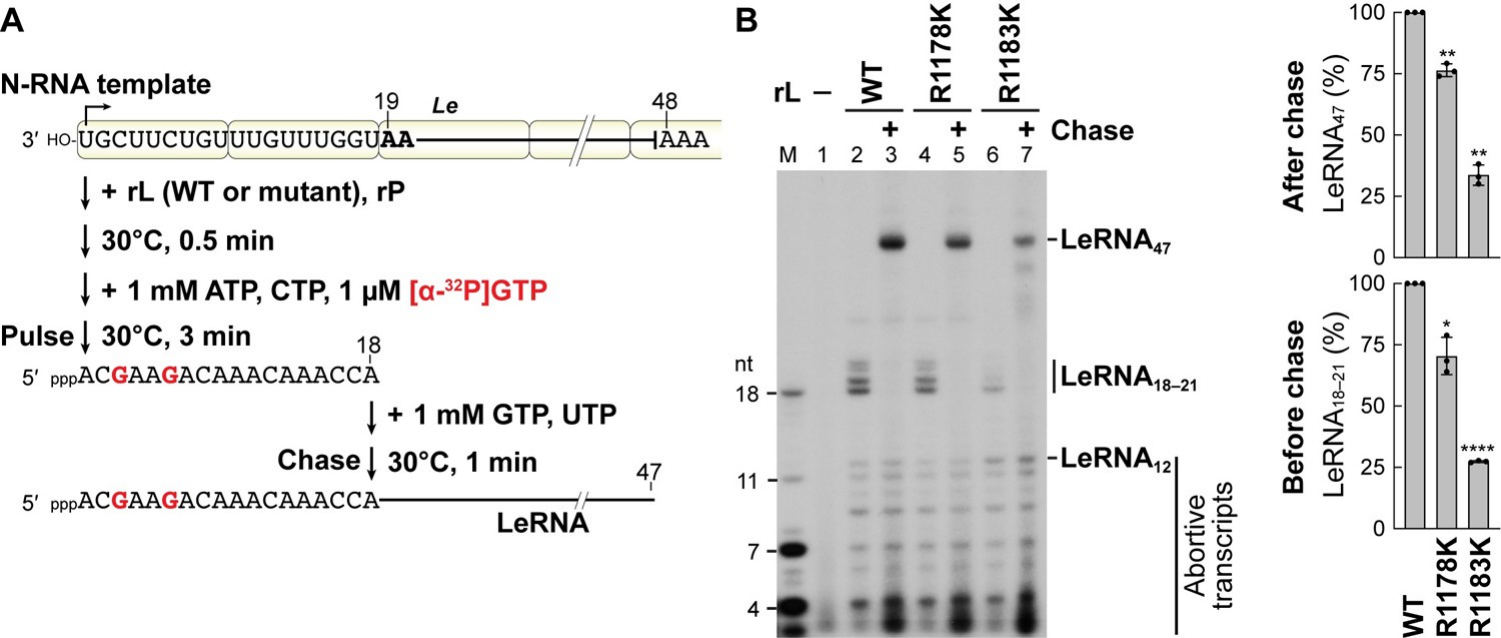
The R1183K mutation diminishes the transition from an early elongation phase to a productive elongation phase of LeRNA synthesis. (**A**) The recombinant VSV L (WT or mutant) and P proteins were incubated with the N-RNA template. After addition of ATP, CTP, and [α-^32^P]GTP, the reaction mixtures were incubated for 3 min to form stable elongation complexes containing ^32^P-labeled pre-LeRNA of 18 nt (LeRNA_18_) (pulse). After addition of excess concentrations of GTP and UTP, the reaction mixtures were further incubated for 1 min to elongate LeRNA_18_ into full-length LeRNA of ~47 nt (LeRNA_47_) (chase). (**B**) RNA products before (lanes 1, 2, 4, and 6) and after (lanes 3, 5, and 7) the pulse reactions were analyzed by 20% urea-PAGE followed by autoradiography (left). Lane 1 indicates no L. M lane shows marker RNAs. Relative LeRNA_18–21_ (right, lower) and LeRNA_47_ (right, upper) synthesis activities of the mutants before and after chase, respectively, are expressed as percentages of the activity of the WT L protein. The dot-plots, columns, and error bars represent the individual values, means, and standard deviations, respectively (n = 3). Statistical significance was determined by one-way ANOVA [ns, not significant (p ≥ 0.05); *, p < 0.05; **, p <0.01; ***, p < 0.001; ****, p < 0.0001; compared to control (WT)].

We further analyzed LeRNA chain elongation activities of selected mutants, R1178K and R1183K, using our pulse-chase LeRNA synthesis assay [35] (**Fig 4A**). As reported [35], during the pulse period in the absence of UTP, the WT L protein generated [α-^32^P]GMP-labeled LeRNA with residues 1–18 (LeRNA_18_), some transcripts longer than expected (19–21 nt), and abortive transcripts (3–12 nt) (**Fig 4B**, lane 2). By adding excess concentrations of GTP and UTP after the pulse reaction, the WT L protein elongated [α-^32^P]GMP-labeled LeRNA_18_ into full-length LeRNA (47 nt) (lane 3). Similarly, the R1178K mutant produced LeRNA_18_ (lane 4) and elongated it to full-length LeRNA (lane 5) during the pulse and chase periods, respectively, although its LeRNA synthesis activity was slightly lower than that of the WT L protein. As in the case of the WT L protein, the R1183K mutant produced abortive transcripts with 3–12 nt during the pulse period (lane 6). However, the R1183K mutant produced lower and higher amounts of LeRNA_18_ and LeRNA_12_, respectively (lane 6), by comparison to the WT L protein (**S1C Fig**). Interestingly, once LeRNA_18_ was formed (**Fig 4B**, lane 6), the R1183K mutant was found to elongate it to full-length LeRNA during the chase period (lane 7). Since the R1183K mutation did not affect terminal *de novo* initiation as shown in Fig 3D (lane 9), it seems likely that R1183K mutation represses an early phase of LeRNA chain elongation, such as escape from the 3′-terminal *Le* promoter followed by the formation of a stable LeRNA elongation complex.

### The R1178 residue is required for efficient polyadenylation-coupled termination at gene-junctions

**Fig 5:**
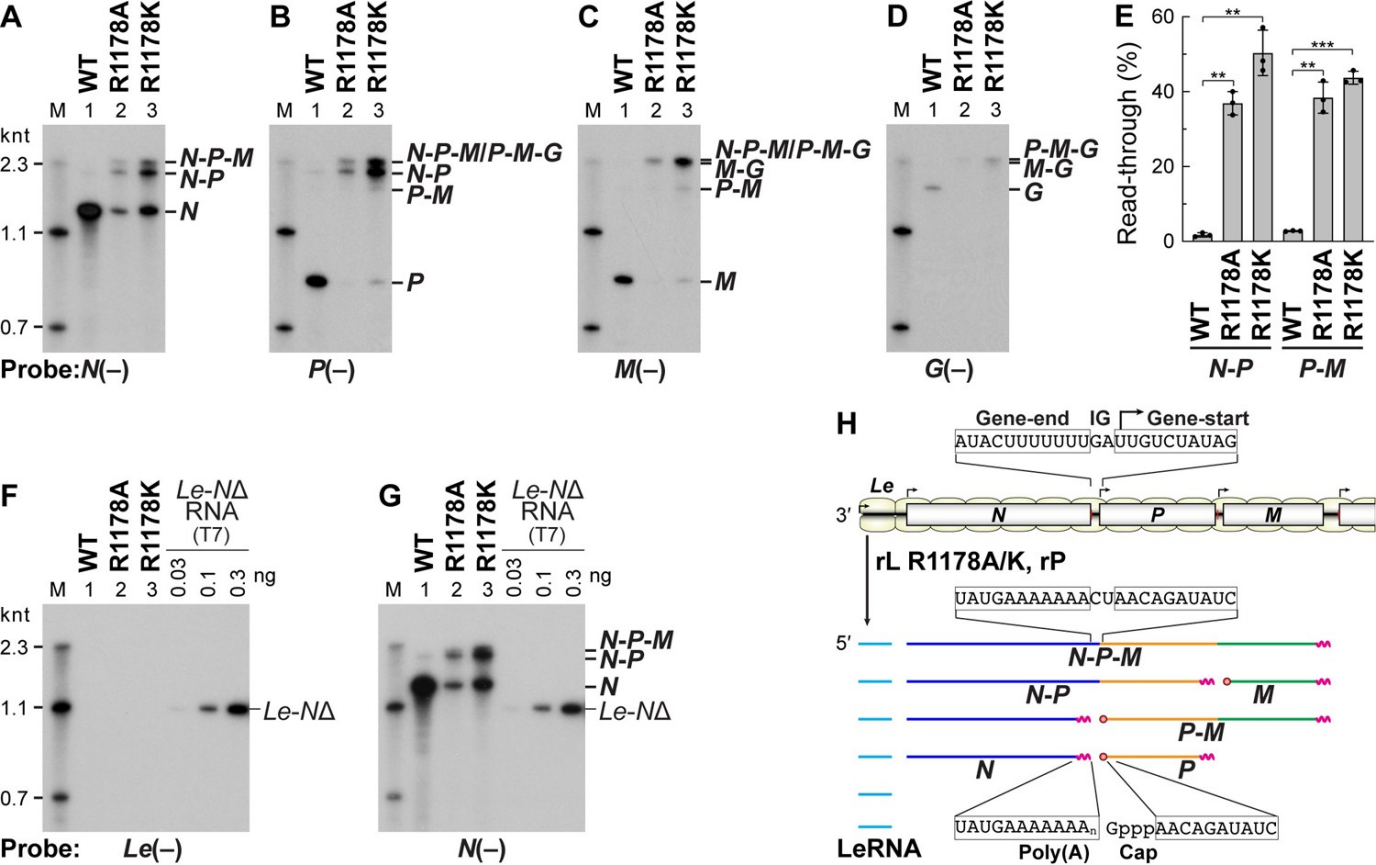
The R1178 mutations trigger the production of polycistronic mRNAs by diminishing polyadenylation-coupled termination at the gene-end sequences. mRNAs were synthesized with the WT or mutant L protein, and analyzed by 5% urea-PAGE followed by Northern blotting using ^32^P-labeled oligo-DNA probes complementary to *N* (**A** and **G**), *P* (**B**), *M* (**C**), and *G* (**D**) mRNAs and positive-strand leader (*Le*) region (**F**) (named *N*(−), *P*(−), *M*(−), *G*(−), and *Le*(−), respectively). M lanes indicate RNA size markers. (**E**) Percentages of read-through at the *N*-*P* and *P*-*M* junctions were calculated by densitometric analysis of band intensities of the terminated and read-through mRNAs on the *N* (**A**) and *P* (**B**) blots, respectively. The dot-plots, columns, and error bars represent the individual values, means, and standard deviations, respectively (n = 3). Statistical significance was determined by one-way ANOVA [ns, not significant (p ≥ 0.05); *, p < 0.05; **, p <0.01; ***, p < 0.001; ****, p < 0.0001; compared to control (WT)]. In panels (**F**) and (**G**), an RNA containing the *Le* region and a part of *N* region (*Le*-*N*Δ) was synthesized by T7 RNA polymerase and used as a positive control. (**H**) A schematic diagram of transcripts (lower) synthesized from the genome (upper) by the R1178 mutants is shown. The *Le*, *N*, *P*, and *M* regions in transcripts are colored in cyan, blue, orange, and green, respectively. Partial nucleotide sequences of the genome and transcripts are shown. IG indicates the intergenic sequence.

In order to identify the long transcripts synthesized by the R1178A and R1178K mutants, Northern blotting was performed with ^32^P-labeled oligo-DNA probes complementary to *N*, *P*, *M*, and *G* mRNAs [named *N*(−), *P*(−), *M*(−), and *G*(−), respectively]. As reported previously [23,34], these probes specifically detected 1.3-knt *N* (**Fig 5A**, lane 1), 0.8-knt *P* (**Fig 5B**, lane 1), 0.8-knt *M* (**Fig 5C**, lane 1), and 1.6-knt *G* (**Fig 5D**, lane 1) monocistronic mRNAs as major products synthesized by the WT L proteins. In contrast, in the cases of transcripts generated by the R1178 mutants, the *N*(−) probe detected multiple transcripts, such as 1.3-knt *N* mRNA, 2.1-knt RNA, and ~3-knt RNA (**Fig 5A**, lanes 2 and 3), in which the latter two RNAs were also reacted with the *P*(−) probe (**Fig 5B**, lanes 2 and 3). Furthermore, ~3-knt RNA were hybridized with the *M*(−) probe as well (**Fig 5C**, lanes 2 and 3). These data indicate that these 2.1-knt and ~3-knt RNAs are *N*-*P* and *N*-*P*-*M* polycistronic mRNAs, respectively. Similarly, *P*-*M* and *N*-*P*-*M*/*P*-*M*-*G* polycistronic mRNAs could be detected in transcripts synthesized by the R1178K mutant either with the *P*(−) (**Fig 5B**, lane 3) or *M*(−) (**Fig 5C**, lane 3) probe. *P*-*M*-*G* and/or *M*-*G* polycistronic mRNAs generated by the R1178K mutant appeared to be reacted with the *G*(-) probe (**Fig 5D**, lane 3). The percentages of read-through at the *N*-*P* and *P*-*M* junctions by the R1178A and K mutant L proteins were 34–57%, whereas those by the WT L protein were 1–3% (**Fig 5E**). To examine whether *Le*-*N* read-through products are generated by the R1178 mutants, Northern blotting were carried out with a probe complementary to LeRNA [*Le*(−)] (**Fig 5F**). Although a positive control RNA containing the *Le* and *N* regions synthesized by T7 RNA polymerase (*Le*-*N*Δ) was detected with the *Le*(−) probe, any transcripts synthesized by the R1178 mutants (lanes 2 and 3) as well as the WT L protein (lane 1) were not reacted with this probe. In contrast, when the same samples were re-probed with the *N*(−) probe (**Fig 5G**), transcripts containing the *N* region were detected (lanes 1–3) as shown in **Fig 5A**. These results indicate that the R1178 mutants frequently read through the gene-junctions, but not the *Le*-*N* junction (**Fig 5F**). We analyzed sequences of the *N*-*P* junction in 12 cDNA clones derived from polyadenylated polycistronic mRNAs synthesized by the R1178K mutant L protein and confirmed that all the clones contain no extra A residues introduced in the junction (**S2 Fig**), indicating that the polycistronic mRNAs are *bona fide* read-through products (**Fig 5H**).

**Fig 6:**
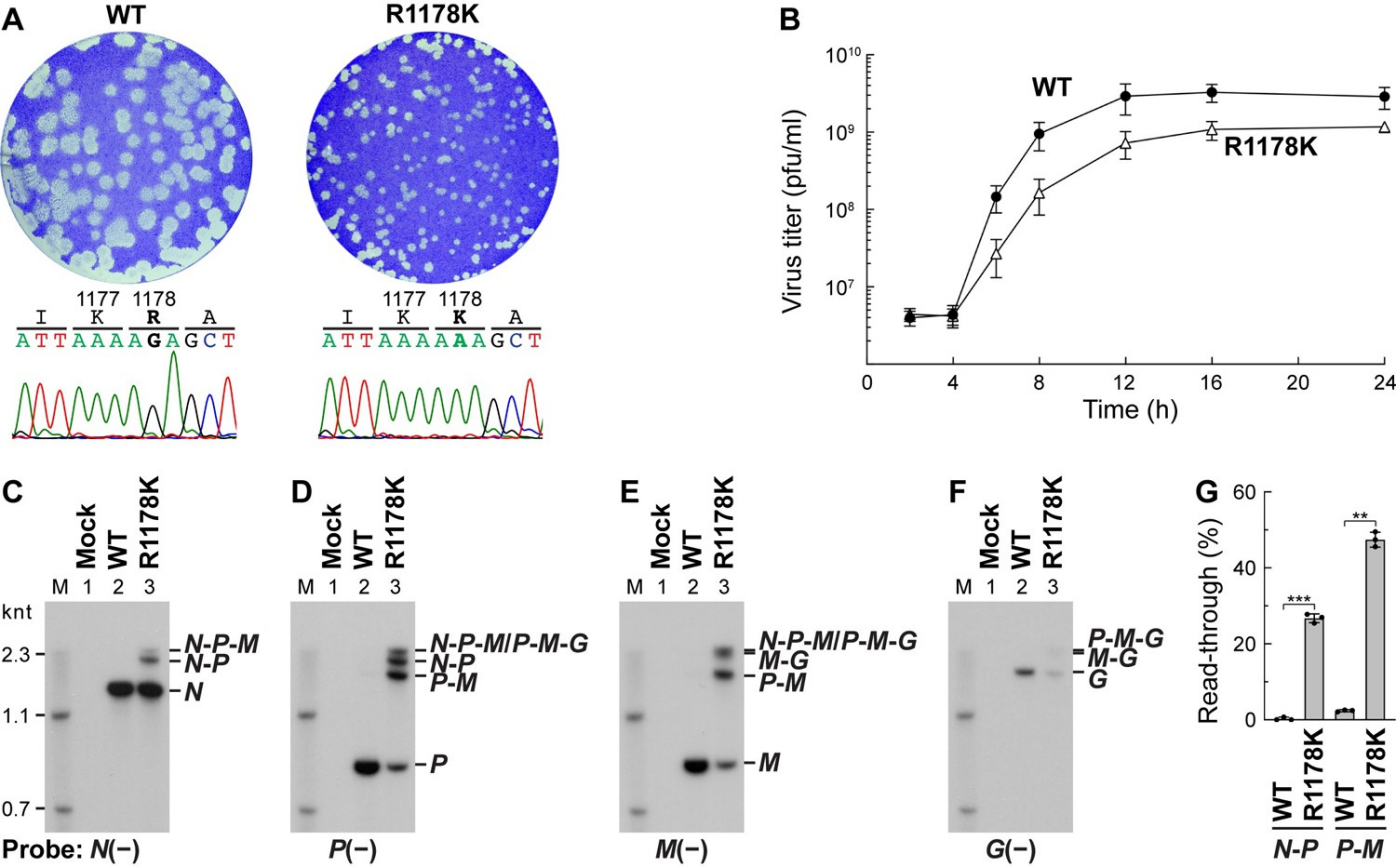
Recombinant VSV with the R1178K mutation in the *L* gene produces polycistronic mRNAs in infected cells. (**A**) Recombinant (r) VSV with the R1178K mutation was generated from cDNA and its plaque phenotype (right) was compared with that of WT rVSV (left). Nucleotide sequences of the mutation sites in genomes of plaque-purified rVSVs are shown with their corresponding amino acid sequences. (**B**) Single-step growth curve experiments were performed by infecting BHK-21 cells with rVSV harboring the WT (closed circles) or R1178K (open triangles) *L* gene at a multiplicity of infection of 5. Virus titers are expressed as plaque-forming unit (pfu) per ml. Symbols and error bars represent the means of titers and standard deviations, respectively (n = 3). (**C–F**) Total RNAs were extracted from mock-, WT rVSV-, or R1178K mutant rVSV-infected cells at 9-h post-infection and treated with RNase H in the presence of oligo(dT). The RNase H-treated RNAs (mock, 0.1 μg; WT, 0.1 μg; R1178K, 0.01 μg) were analyzed by Northern blotting using the indicated probes as described in Fig 5. (**G**) Percentages of read-through at the *N*-*P* and *P*-*M* junctions were calculated as in Fig 5E. Statistical significance was determined by Student’s t test [ns, not significant (p ≥ 0.05); *, p < 0.05; **, p <0.01; ***, p < 0.001; ****, p < 0.0001; compared to control (WT)].

Using the VSV reverse genetics system [36], we examined the effects of the R1178K and R1183K mutations in the *L* gene of the VSV genome on generation and growth of recombinant mutant VSVs in host cells. However, VSV harboring the R1183K mutation could not be recovered, although we tried multiple times, suggesting that the R1183K mutation is lethal to VSV. On the other hand, VSV with L mutation R1178K was successfully generated, but exhibited a small-plaque phenotype (**Fig 6A**, right upper) when compared to the WT virus (left upper). We confirmed that the *L* gene from plaque-purified R1183K mutant VSV has the R1183K mutation (**Fig 6A**, right lower), but not any other nucleotide changes in the *N*, *P*, and *L* genes. Single-step growth curve experiments showed that the R1178K mutant is grown more slowly than the WT virus (**Fig 6B**). Unexpectedly, the R1178K mutant produced 5–10-fold higher levels of *N*, *P*, *M*, and *G* monocistronic mRNAs than the WT virus at 6-h, 9-h, and 12-h post-infection (**S3 Fig**). On the other hand, levels of the N, P, and G proteins expressed in the R1178K mutant virus-infected cells were slightly higher than those in the WT virus-infected cells, while levels of the M protein in the R1178K mutant virus-infected cells were equivalent or lower than those in the WT virus-infected cells (**S4 Fig**). Although, it is, at present, not clear whether all these unique phenotypes of the mutant virus are caused by the R1178K mutation alone and/or another unidentified complementation mutation(s) in the genome, we observed that the mutant virus produced large amounts of polycistronic mRNAs in addition to the monocistronic mRNAs throughout the course of infection (**S3 Fig**) as expected. To identify these polycistoronic mRNAs, we performed Northern blotting with the probes against *N* (**Fig 6C**), *P* (**Fig 6D**), *M* (**Fig 6E**), and *G* (**Fig 6F**) mRNAs. Since the amount of the monocistronic *N* mRNA accumulated in R1178K mutant VSV-infected cells at 9-h post-infection were approximately 10-fold higher than that in WT VSV-infected cells (**S3A Fig**), a 10-fold lower amount of the total RNAs from R1178K mutant-infected cells than those from WT virus-infected cells were analyzed. The WT virus mainly generated monocistronic mRNAs (**Fig 6C–6F**, lane 2), whereas R1178K mutant VSV was found to synthesize polycistronic read-through mRNAs, such as *N*-*P*, *N*-*P*-*M*, and *P*-*M*, in addition to monocistronic mRNAs (lane 3) as observed in *in vitro* transcription. The percentages of read-through at the *N*-*P* and *P*-*M* junctions by the R1178K mutant virus were 27% and 48%, respectively, whereas those by the WT virus were 0.3% and 2%, respectively (**Fig 6G**), indicating that the R1178K mutation significantly increases read-through at the gene junctions in infected cells.

## Discussion

We have previously demonstrated that the conserved tryptophan residue (W1167) on the priming-capping loop extended from the PRNTase domain of the VSV L protein is critical for *de novo* initiation at the 3′-terminal *Le* promoter to generate the AC dinucleotide [29]. However, it remains largely unknown how the L protein changes the mode from initiation to elongation during LeRNA synthesis. Similar to prokaryotic and eukaryotic DNA-dependent RNA polymerases, the VSV L-P RdRp complex produces high levels of abortive transcripts (3–12 nt) from the *Le* promoter. However, once nascent transcripts reach their lengths of >12 nt, the RdRp seems to escape from the *Le* promoter to enter a productive elongation phase by establishing a stable elongation complex. We have previously shown that ~18-nt nascent LeRNA associated with such stable elongation complexes can be efficiently extended to the full-length LeRNA (47 nt) [35]. In this study, we found that the R1183 mutations (R1183A and R1183K) negatively impact the transition from an early elongation phase to a productive elongation phase of LeRNA synthesis, but not terminal *de novo* initiation or productive elongation (**Figs 3** and **4**). Synthesis of full-length LeRNA is a prerequisite for transcription initiation from the internal *N* gene-start sequence [5]. The R1183K mutant could not synthesize *N* mRNA efficiently, presumably because it might not be able to reach the *N* gene-start sequence due to the deficiency in the *Le* promoter escape. Consistent with these observations, rVSV harboring the R1183K mutation could not be recovered by the reverse genetics system, suggesting that this mutation is lethal to VSV replication in cultured cells.

Although the precise role of R1183 in LeRNA synthesis currently remains elusive, our finding provides the first insight into the regulatory mechanism of the *Le* promoter escape with the specific residue in the PRNTase domain of the transcribing L protein. Since R1183 is far from the RdRp active site (~54 and 45Å to D605 and D714, respectively), there appears to be a unique mechanism to regulate the *Le* promoter escape. In the structure of the VSV L protein complexed with an N-terminal fragment of the P protein [37], one of the ζ-amino groups in the side chain of R1183 sits within 3.3Å of the mainchain carbonyl group of H1217. This residue or it’s associated loop could play a structural role with the polymerase. The catalytic HR motif of the PRNTase resides downstream on this same loop structure. However, the side chain of R1183 (**Fig 2**) as well as that of H1217 [17] is not essential for the PRNTase activity of the VSV L protein. Furthermore, R1183 is not required for terminal *de novo* initiation at the *Le* promoter (**Fig 3D**). In the apo-structure, the basic helix structure as well as the active site of the PRNTase domain is covered with the connector domain, which is linked to the C-terminal end of the PRNTase domain via a flexible linker [37]. The positively-charged guanidium group of R1183 is within ~6 Å of the side chains of several residues on the linker but oriented away from these residues, suggesting that R1183 is not bound in the apo-state of the L protein. However, with simple side chain rotamer rotations of R1183, E1339 and Q1342, R1183 would be within bonding distance of E1339 or Q1342. To shift between different states of polymerase activity, movements both locally and movement of the connector domain, specifically, and other C-terminal accessory domains, in general, is required. These states may also require transient stabilization. In this case, R1183 could play this role by tethering to the linker through interactions with E1339 or Q1342, or with other residues that were not resolved in the current reconstruction (e.g., 1333–1338) (**S5 Fig**).

After terminal *de novo* initiation, the priming-capping loop may undergo dynamic structural rearrangements to open a putative transcript exit channel, which may be located between the PRNTase and RdRp domains. Nascent LeRNA emerging from the putative exit channel may serve as a driving force for the exclusion of the priming-capping loop from the RdRp active site cavity followed by its flipping toward the PRNTase active site, as observed for HRSV (**Fig 1C**). Simultaneously, the connector domain should be dissociated from the PRNTase domain to make a space for the rearranged priming-capping loop as well as the elongating LeRNA. In the putative post-initiation state of the VSV L protein (**Fig 1B**), which was modeled based on the apo-state of the HRSV L protein [31], the flipped loop infringes on the space occupied by the connector domain in the apo-structure of the L protein. Thus, the displacement of the connector domain with the flipped position of the priming-capping loop may make R1183 accessible to its ligand(s), if any. It is possible that sensing of the emerging LeRNA or rearranged priming-capping loop with R1183 may trigger a further conformational change of the L protein, allowing the transition from the early elongation phase to the productive elongation phase of LeRNA synthesis. We speculate that this transition step is required to establish a stable elongation complex on the N-RNA template around position 18 from the 3′-end of the *Le* promoter.

In this study, we also found that the mutations of R1178 (R1178A and R1178K) significantly reduced the efficiency of stop-start transcription at the gene-junctions, but not at the *Le*-*N* junction, resulting in the production of larger amounts of polycistronic mRNAs than the WT L protein (**Figs 5** and **6**). We also confirmed that *N*-*P* polycistronic mRNAs do not contain any additional A residues at the *N*-*P* junction (**S2 Fig**). Therefore, the R1178K mutation may interfere with efficient recognition of the gene-end sequences in the genome or their complementary sequence in transcripts during transcription, inhibiting polyadenylation-coupled termination of mRNA synthesis.

**Fig 7:**
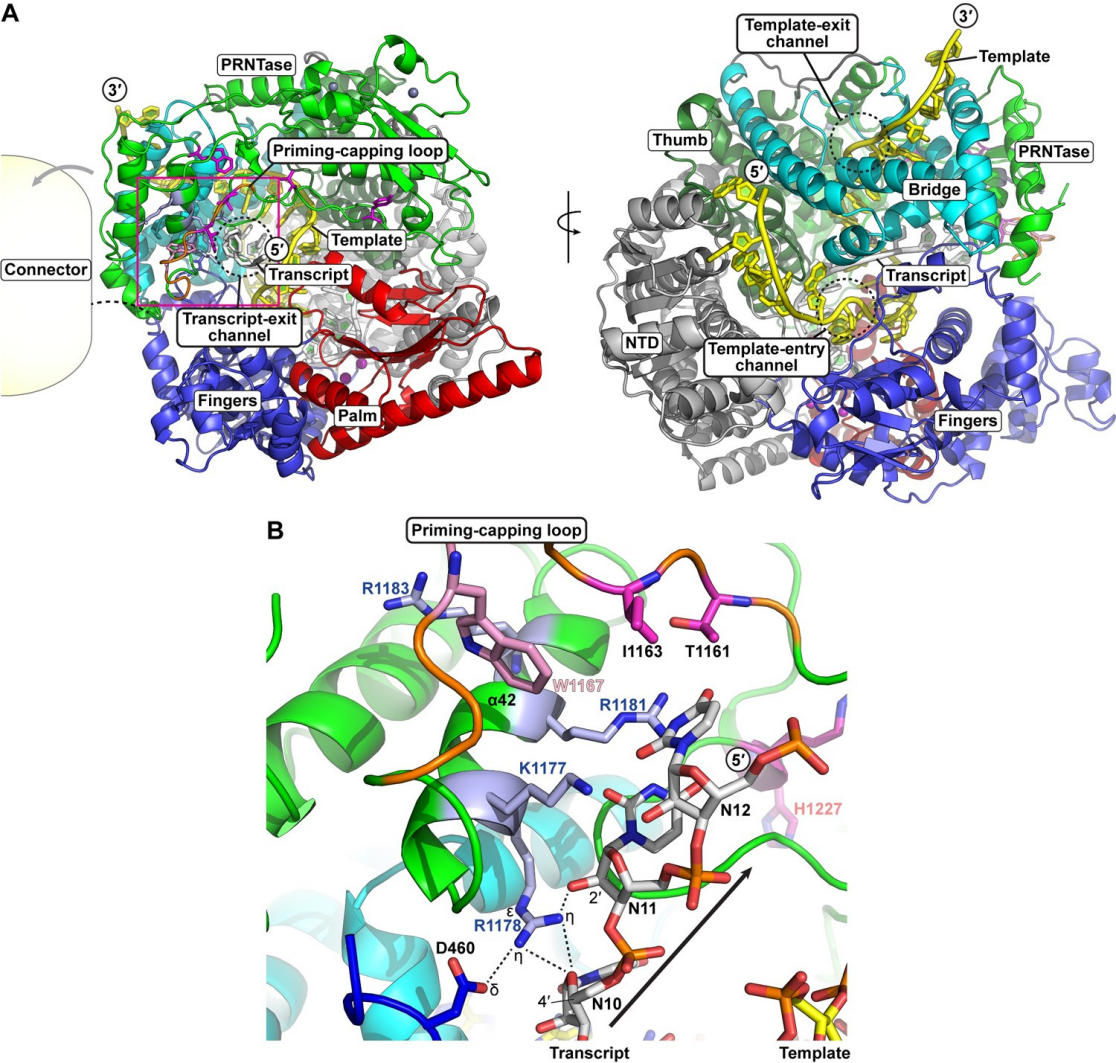
Model of a putative elongation complex of the VSV L protein. (**A**) Two views of a modeled VSV L RdRp core structure (residues 35–1332) in an elongation state (Model Archive id: ma-ecf5b) are shown as cartoon models with the N-terminal, fingers, palm, thumb, bridge, and PRNTase subdomains colored in gray, blue, red, dark green, cyan, and green, respectively. Poly(A) and poly(U) strands of RNA, analogous to the template and emerging RNA strand, are shown in stick models with yellow and off-white shading. The priming-capping loop is shaded with a background of orange. Key amino acid residues required for capping (magenta), the priming residue (pink), and the basic residues (light blue) of helix α42 are shown in ball and stick representation. Proximity of the connector domain is shown with the yellow bubble to the left. In (**B**), a close-up of residues in the putative exit tunnel for transcription/replication products is shown. The side chain of residue R1178 is within bonding distance of the 2′-OH and 4′-oxygen of adjacent ribosyl group with the exiting RNA, while also partially bonding to one of the δ-oxygens of residue D460.

To speculate a role of R1178 in transcription, we modeled a structure of a putative elongation complex of the VSV L protein based on rotavirus VP1 RdRp ([38], PDB id: 6OJ6) (**Fig 7A**). In the RdRp active site cavity of the modeled VSV elongation complex, a 12-base transcript is extended from the RdRp active site on the palm subdomain toward the luminal side of the α-helical region (residues 940–1072, referred to as "bridge" in [1]), a region that is functionally unknown. The bridge region is anchored to the C-terminal end of the proposed RdRp thumb subdomain (residues 789–932) via an unstructured loop. A part (e.g., α36, residues 1011–1020) of this subdomain contacts the PRNTase (sub)domain and, thus, may act as a scaffold for the PRNTase. A 23-base segment of RNA representing the template strand threads into and out of the polymerase, where it is partially paired with the transcript, to form a template-transcript hybrid. At the luminal side of the bridge region, the template-transcript hybrid is split into single stranded RNAs, which exit from the lumen through different channels. In the model, the bridge region is predicted to serve as a template exit channel (**Figs 7A**, right; **S6**). Regarding the newly formed transcript, a transcript exit channel appears between the fingers subdomain and the PRNTase domain with a flipped priming-capping loop displaced from the channel (**Fig 7A**, left). Though the C-terminal domains were not modeled in this complex, the connector domain is likely to be displaced by the emerging priming-capping loop and/or the emerging transcript, further intriguing the role of R1183.

In the modeled structure of the elongation complex, the basic side chain of R1178 points to a lumen of the putative transcript exit channel. In both the apo- and elongation models, the *N*^ε^-hydrogen of the R1178 side chain bonds to the backbone carbonyl oxygen of the serine 1019 (bridge subdomain). For the rest of the guanidinium group of R1178, the *N*^η^-hydrogens have contact with the ribose backbone of a modeled transcript at the 10^th^ and 11^th^ nucleotide positions from the RdRp active site in the elongation state (**Fig 7B**), while one of the *N*^η^-hydrogens contacts one of the δ-oxygens in the side chain of D460 (fingers subdomain). In the apo-state, one of the *N*^η^-hydrogens is bound to the backbone carbonyl oxygen of V1173, potentially stabilizing the conformation of the priming-capping loop in the down position (**S7 Fig**). Finally, in this apo-state, the *N*^η^-hydrogen has split coordination with the γ-oxygen and the carbonyl group of S1019 (same coordination as the *N*^ε^-hydrogen). Mutation of R1178 to either alanine or lysine, would modulate the coordination of this residue both as a structural element, as well as, how it interacts with the exiting RNA. Based on this model (**Fig 7B**), we speculate that R1178 may regulate polyadenylation-coupled termination at the gene-end sequences by interacting with elongating transcripts rather than the template. A lack of interaction with the exiting transcripts, as with R1178K and R1178A, could unattenuate the speed of RNA production or exit, affecting polyadenylation-coupled termination of mRNA synthesis. On the other hand, the R1178 residue does not play any roles in termination of LeRNA synthesis at the *Le*-*N* junction, suggesting that the mechanism of termination of LeRNA synthesis is different from that of polyadenylation-coupled termination of mRNA synthesis at the gene-junctions. However, it is currently not clear how the proposed interaction of R1178 with exiting transcripts induces stuttering of the RdRp domain at the poly(U) stretch in the gene-end sequence to add a poly(A) tail to their 3′-ends. It is interesting to note that similar read-through phenotypes of HRSV are associated with the M1169V [39] and N1049D [40] mutations in the bridge region of the L protein, which may serve as a template exit channel of the RdRp domain.

One of the interesting observations in this study is that the R1178A mutant prefers GTP rather than GDP as the pRNA acceptor substrate, resulting in the formation of a larger amount of GppppA than GpppA (**Fig 2B**). It seems likely that the R1178A mutation may distort the pRNA acceptor binding site of the PRNTase domain into a conformation that can flexibly bind GTP as well as GDP, but not disrupt the active site of the enzyme. In contrast, the R1178K mutant exhibited the same acceptor substrate specificity as the WT L protein, indicating that the basic nature of the residue is required for the preferential recognition of GDP to generate GpppA. Nevertheless, the R1178K mutant as well as the R1178A mutant is not able to conduct stop-start transcription at the gene junctions properly, suggesting that arginine at this position plays a critical role in polyadenylation-coupled termination at the gene-end sequence to produce monocistronic mRNAs. On the other hand, arginine at position 1183 of the VSV L protein is also obligatory for the *Le* promoter escape. Thus, it would be interesting to investigate roles of other rhabdoviral counterparts of these basic amino acid residues in their RNA biosynthesis.

Here, we identified two unique mutations in the PRNTase domain of the VSV L protein that prevent *Le* promoter escape or polyadenylation-coupled termination at the gene-end sequences during stop-start transcription. These observations strongly suggest that the PRNTase domain plays multiple roles in conducting accurate stop-start transcription beyond its known role in pre-mRNA capping. Further biochemical and structural studies are necessary to provide an overall picture of transcriptional control with the PRNTase domain of rhabdoviruses and other NNS RNA viruses.

## Materials and methods

### VSV proteins

The recombinant VSV L and P proteins were expressed as His-tagged proteins in Sf21 insect cells and purified as described previously [16,20,34]. Site-directed mutagenesis was performed to generate mutant L proteins as described in [29]. GST and GST-fused P protein were expressed in *E*. *coli* and purified as described previously [29]. Purified proteins were analyzed by electrophoresis in polyacrylamide gels containing SDS (SDS-PAGE) followed by staining with One-Step Blue (Biotium). The N-RNA template was isolated from purified VSV particles as described in [16].

### Recombinant VSVs

Recombinant (r) VSVs with the WT or R1178K mutant *L* gene were generated using the reverse genetics system [36,41] and plaque-isolated as described in [23,29]. The *N*, *P*, and *L* genes in the plaque isolated virus particles were sequenced as described in [23]. Single-step growth curve experiments were carried out as described in [29].

### *In vitro* enzyme assays

All the *in vitro* enzyme assays used in this study were described in detail previously [16,24,29,34]. Briefly, *in vitro* oligo-RNA capping was performed with purified L [wild-type (WT) or mutant, 0.06 μg], [α-^32^P]GTP (PerkinElmer), and pppAACAG oligo-RNA for 2 h, as described in [16,34]. After digestion of capped RNA products with calf intestine alkaline phosphatase (CIAP) and nuclease P1, liberated cap structures were analyzed by thin layer chromatography (TLC) on a polyethyleneimine (PEI) cellulose plate followed by autoradiography. When [α-^32^P]GDP was used as a substrate instead of [α-^32^P]GTP, capping reaction mixtures were analyzed by 20% polyacrylamide gels containing 7 M urea (urea-PAGE) followed by autoradiography [24]. The GTPase assay was carried out with L (WT or mutant, 0.3 μg) for 2 h using [α-^32^P]GTP as a substrate [16,20].

*In vitro* transcription was performed with L (WT or mutant, 0.15 μg), P (0.05 μg), and N-RNA template (0.4 μg protein) in the presence of [α-^32^P]GTP and the other three NTPs for 2 h as detailed previously [21,34]. mRNAs were deadenylated with RNase H in the presence of oligo(dT) [34]. ^32^P-Labeled LeRNA and deadenylated mRNAs were analyzed along with RNA size markers by 20% and 5% urea-PAGE, respectively, followed by autoradiography. ^32^P-labeled size marker RNAs (2,346, 1,065, and 670 nt) were prepared by T7 RNA polymerase as described previously [34]. RNA Century-Plus Marker Templates and Decade RNA Marker System (Ambion) were used to prepare ^32^P-labeled long and short RNA ladders, respectively.

*In vitro* AC synthesis was carried out with L (WT or mutant, 0.15 μg), P (0.04 μg), and N-RNA template (0.4 μg protein) in the presence of ATP and [α-^32^P]CTP for 1 h [29]. CIAP-resistant products were analyzed by 20% urea-PAGE as described in [29]. The *in vitro* pulse-chase LeRNA synthesis assay was conducted as in [35]. Briefly, ^32^P-labeled LeRNA_18_ was synthesized with L (0.15 μg), P (0.05 μg), and N-RNA (0.4 μg) in the presence of ATP, CTP, and [α-^32^P]GTP during the 3-min pulse period, and then chased into LeRNA_47_ by incubating with excess concentrations of GTP and UTP for 1 min. Transcripts were analyzed along with RNA size markers (synthesized by T7 RNA polymerase) by 20% urea-PAGE as described in [35].

### GST pull-down assay

The GST pull-down assay was performed with purified GST-fused P protein and the His-tagged L protein (WT or mutant, 1 μg) as described in [29].

### Northern blotting

Unlabeled transcripts were synthesized with L (WT or mutant, 0.15 μg), P (0.05 μg), and N-RNA template (0.4 μg protein) *in vitro*, and deadenylated as described in [34]. One tenth amounts of the samples were electrophoresed together with ^32^P-labeled marker RNAs [34] in a 5% denaturing polyacrylamide gel, and transferred from the gel to an Immobilon NY^+^ membrane (Millipore) as described previously [34]. The transcripts on the membranes were probed and re-probed with 5′-end-^32^P-labeled oligo-DNAs complementary to *N*, *P*, *M*, and *G* mRNAs and LeRNA [called *N*(−), *P*(−), *M*(−), *G*(−), and *Le*(−), respectively] as described in [34,42]. An RNA (named *Le*-*N*Δ, 0.03–0.3 ng) containing a 5′-part of the VSV anti-genomic RNA sequence (positions 1–1,034) was synthesized from the pVSVFL-2 plasmid [41], which had been linearized within the *N* gene by BstZ17I, by T7 RNA polymerase and used as a positive control for Northern blotting with the *Le*(−) and *N*(−) probes.

BHK-21 cells (10^6^ cells/well/12-well plate) were infected with WT or R1178K mutant VSV at a multiplicity of infection of 5 or mock-infected and cultured at 37°C. At 6-h, 9-h, and 12-h post-infection, total RNAs were isolated from the infected cells using the Direct-zol RNA Kit (Zymo Research) according to the manufacturer’s instruction. Polyadenylated RNAs in the total RNAs (1 μg) were annealed to oligo(dT)_18_ (0.1 μg) and incubated with 0.5 units of RNase H (Roche Diagnostics) at 37°C for 2 min. The RNase H-treated total RNAs (0.1 or 0.01 μg, indicated in figure legends) were analyzed by Northern blotting as described above. Cellular 18S rRNA was detected using a 5′-^32^P-labeled antisense oligo-DNA probe: 5′-TGG TCG GAA CTA CGA CGG TAT CTG ATC G.

Band intensities of individual monocistronic and polycistronic mRNAs on the *N* and *P* blots were measured using the ImageJ program (National Institutes of Health [43]) and percentages of read-through at the *N*-*P* and *P*-*M* gene junctions were calculated.

### Western blotting

BHK-21 cells (10^6^ cells/well/12-well plate) were infected with WT or R1178K mutant VSV or mock-infected as described above. At 6-h, 9-h, and 12-h post-infection, cells were lysed in 200 μl of an ice-cold lysis buffer [50 mM Tris-HCl (pH 8.0), 150 mM NaCl, 1 mM EDTA, 1% Triton X-100] containing Halt Protease Inhibitor Cocktail (Thermo Scientific). After centrifugation of the cell lysates at 15,000 × *g* for 5 min at 4°C, the resulting supernatants were collected. Protein concentrations of the lysates were determined by a Bradford protein assay (Bio-Rad) using bovine γ-globulin as a standard. Proteins (1 μg) in the lysates were resolved by 10% SDS-PAGE (acrylamide-bisacrylamide ratio of 75:1) and electroblotted onto polyvinylidene difluoride membranes (Millipore, Immobilon P). The membranes were probed with rabbit anti-VSV (Imanis Life Sciences, REA005, 1:10,000 dilution), -P ([16], 1:4,000), or -GAPDH (Invitrogen, PA116777, 1:500) polyclonal antibody followed by horseradish peroxidase-conjugated goat anti-rabbit IgG polyclonal antibody (Invitrogen, 32460). The immuno-complexes were detected using SuperSignal West Dura Extended Duration Substrate (Thermo Scientific) according to the manufacturer’s instruction. The membranes were treated with Restore PLUS Western Blot Stripping Buffer (Thermo Scientific) and re-probed with another antibody. Band intensities were measured using the ImageJ program [43].

### Statistical analysis

All the experiments using the *in vitro* enzyme assays were repeated three times with the same enzyme preparations. Northern and Western blot analyses were repeated three times with different sample preparations. Statistical analyses were performed by one-way analysis of variance (ANOVA) or Student’s t test using the Graphpad Prism software (ver. 9.3).

### Sequence analysis of *N*-*P* read-through products produced by the R1178K mutant L protein

Unlabeled transcripts were synthesized with the R1178K mutant *in vitro* as described above. After removing N-RNA from the reaction mixture by ultracentrifugation [23], transcripts were purified by Direct-zol RNA Kit. First-strand cDNAs were synthesized from the purified transcripts with SuperScript IV reverse transcriptase (Thermo Fisher Scientific) using an oligo(dT)_20_ primer, and subjected to PCR with Q5 polymerase (New England Biolabs) to amplify the *N*-*P* junction using the following primers: GGA ATT CGC AGG TTT GTT GTA CGC TTA TG (EcoRI site underlined) and GGT AAG CTT TCC TCA TCT GCA TAG TCA TCT AAA G (HindIII site underlined). The resulting PCR products were cloned into the EcoRI and HindIII sites of the pGEM-3Z plasmid (Promega) and sequenced.

### WebLogo sequence analysis

Local amino acid sequences of 109 rhabdoviral L proteins were analyzed by WebLogo program [44] (http://weblogo.berkeley.edu/) as described in [29].

### Modeling of partial structures of the VSV L protein in post-initiation states

The protein coordinates corresponding to the VSV L (PDB id: 6U1X, [37]) were downloaded from the RCSB [45]. The priming-capping loop and flanking residues of VSV L were modeled in the putative post-initiation state based on spacial position of corresponding residues in HRSV L structure (PDB id: 6PZK, [31]) with SWISSMODEL [46]. The coordinates of helix α42 (residues 1175–1186) in the putative post-initiation state were superimposed by the least-square fitting routine in COOT to the VSV L structure (e.g., 6U1X). Residues 1155–1177 of the superimposed model were replaced into the VSV L model, and the two connections points were regularized. This model (residues 35–1332) was then subjected to energy minimization with YASARA [47]. The structure of a putative elongation complex of the VSV L protein was generated based on the nucleic acid-bound structure of rotavirus VP1 RdRp ([38], PDB id: 6OJ6). The two protein structures were aligned in Coot [48]. Idealized RNA was aligned with nucleic acid in rotavirus structure. Minor adjustments were made with the RNA to avoid collision with the protein. The protein-nucleic acid complex was subjected to energy minimization with YASARA. Structural images were generated using the PyMOL software [49].

## Supporting information

S1 Fig Production of prematurely-terminated LeRNA during *in vitro* transcription. (**A**) The full-length LeRNA (LeRNA_47_) and prematurely-terminated LeRNA with ~12 nt (LeRNA_12_) contain 7 and 2 G residues, respectively, at the indicated positions. (**B**) Based on the radioactivities of [α-^32^P]GMP-labeled LeRNA_47_ and LeRNA_12_ (Fig 3C, lanes 1–9) and the number of the G residues in these RNAs, relative molar amounts of LeRNA_47_ (gray columns, the same as in Fig 3C) and LeRNA_12_ (open columns) synthesized by the WT or mutant L protein during 2-h transcription were estimated. The amount of LeRNA_47_ synthesized by the WT L protein (Fig 3C, lane 2) was set to 100%. Statistical significance for differences in the amounts of LeRNA_12_ synthesis by the mutant L proteins compared to the WT L protein (open column 2) was examined by one-way ANOVA [ns, not significant (p ≥ 0.05); *, p < 0.05; **, p <0.01]. (**C**) Relative molar amounts of LeRNA_18_ (gray columns) and LeRNA_12_ (open columns) synthesized by the WT or mutant L protein during 3-min transcription in the absence of UTP (Fig 4B, lanes 2, 4, and 6) were estimated. The amount of LeRNA_18_ synthesized by the WT L protein (Fig 4B, lane 2) was set to 100%. Statistical significance for differences in the amounts of LeRNA_12_ synthesis by the mutant L proteins compared to the WT L protein (open column 2) was examined by one-way ANOVA.
(TIF)

S2 Fig Sequences of the *N*-*P* junction in *N*-*P* polycistronic mRNAs synthesized by the R1178K mutant L protein. The *N*-*P* junction in 12 cDNA clones derived from polyadenylated polycistronic mRNAs synthesized by the R1178K mutant L protein *in vitro* were sequenced. Electropherograms are shown with the *N*-*P* junction sequence (top). The *N* gene-end and *P* gene-start sequences are boxed. IG indicates the intergenic sequence.
(TIF)

S3 Fig Time course of viral mRNA synthesis in cells infected with the R1178K mutant rVSV. BHK-21 cells were mock-infected or infected with the WT or R1178K mutant rVSV at a multiplicity of infection of 5 and cultured at 37°C. Total RNAs were extracted from the cells at 6-h, 9-h, and 12-h post-infection and treated with RNase H in the presence of oligo(dT). The RNase H-treated RNAs (0.1 μg) were analyzed by Northern blotting sequentially with the probes against *N* (**A**), *P* (**B**), *M* (**C**), and *G* (**D**) mRNAs as in Fig 5. The graphs show relative amounts of monocistronic mRNAs synthesized in cells infected with the WT (closed circles) or R1178K mutant (open triangles) rVSV. The amounts of the viral monocistronic mRNAs in the total RNAs from the WT virus-infected cells at 12-h post-infection was set to 1. Symbols and error bars represent the means and standard deviations, respectively, of three independent experiments (n = 3). (**E**) Cellular 18S rRNA on the same membrane was detected with an antisense probe.
(TIF)

S4 Fig Time course of viral protein synthesis in cells infected with the R1178K mutant rVSV. BHK-21 cells were mock-infected or infected with the WT or R1178K mutant rVSV as in S3 Fig. Cell lysates were prepared at 6-h, 9-h, and 12-h post-infection. (**A**) The cell lysates (1 μg protein) were analyzed by Western blotting with rabbit anti-VSV (N, M, and G proteins), -P, or -GAPDH polyclonal antibody. The graphs show relative amounts of the N, P, M, and G proteins synthesized in cells infected with the WT (closed circles) or R1178K mutant (open triangles) rVSV. The amounts of the viral proteins in the lysates from the WT virus-infected cells at 12-h post-infection was set to 1. Symbols and error bars represent the means and standard deviations, respectively, of three independent experiments (n = 3). (**B**) The cell lysates (10 μg protein) were analyzed by 10% SDS-PAGE followed by staining with Coomassie Brilliant Blue.
(TIF)

S5 Fig Environment of VSV L residue, Arginine-1183. The model of a putative elongation complex of the VSV L protein is shown with colors as in Fig 7. The connector domain (yellow) is placed based on superposition of the modeled polymerase core and with the intact VSV polymerase model (PDB id: 6U1X). R1183 sits on the opposite face of helix α42 by comparison to R1178. The side chain of R1183 faces both a loop within the PRNTase and the linker between the PRNTase and connector domain. Residues between 1211–7 (on the PRNTase loop) and 1332–9 (in the linker) are not resolved in PDB id: 6U1X and are connected by a dashed-line.
(TIF)

S6 Fig Putative template-entry and exit channels of the VSV L protein. The modeled structure of the VSV L elongation complex shown in Fig 7A is viewed from a different angle.
(TIF)

S7 Fig Environment of VSV L residue, Arginine-1178, in absence of RNA. In (**A**), a panned out view of the L protein in absence of RNA, noted as the apo-state in the text, is shown in the context of R1178. Secondary structure elements and key residues (shown as sticks) surrounding R1178 are shown with colors as in Fig 7, though residues S1019 and V1173 are shaded yellow here. (**B**) shows a close-up view of the direct environment of R1178, with local and key functional residues noted. (**C**) shows an alternate stick model for a subset of residues in the vicinity of R1178. Residue interactions with guanidinium group of the R1178 side chain are noted with dashed lines. Carbon-backbone colors, in (**C**), correspond to colors as in (**A**) and (**B**). Models in this figure were generated from coordinates in PDB id: 6U1X. For clarity, the N-terminal, fingers (excluding residues 457–62), palm, and thumb subdomain are not shown.
(TIF)

S1 Data Excel spreadsheet containing all data used to generate graphs. (XLSX)

## References

[1] Ogino T, Green TJ. RNA synthesis and capping by non-segmented negative strand RNA viral polymerases: lessons from a prototypic virus. Front Microbiol. 2019;10:1490. doi: 10.3389/fmicb.2019.01490 31354644 PMC6636387

[2] Colonno RJ, Banerjee AK. A unique RNA species involved in initiation of vesicular stomatitis virus RNA transcription in vitro. Cell. 1976;8(2):197–204. doi: 10.1016/0092-8674(76)90003-9 183891

[3] Colonno RJ, Banerjee AK. Complete nucleotide sequence of the leader RNA synthesized in vitro by vesicular stomatitis virus. Cell. 1978;15(1):93–101. doi: 10.1016/0092-8674(78)90085-5 212201

[4] Testa D, Chanda PK, Banerjee AK. Unique mode of transcription in vitro by Vesicular stomatitis virus. Cell. 1980;21(1):267–75. Epub 1980/08/01. 0092-8674(80)90134-8 [pii]. doi: 10.1016/0092-8674(80)90134-8 . 6250715

[5] Emerson SU. Reconstitution studies detect a single polymerase entry site on the vesicular stomatitis virus genome. Cell. 1982;31(3 Pt 2):635–42. Epub 1982/12/01. 0092-8674(82)90319-1 [pii]. doi: 10.1016/0092-8674(82)90319-1 . 6297777

[6] Abraham G, Rhodes DP, Banerjee AK. The 5’ terminal structure of the methylated mRNA synthesized in vitro by vesicular stomatitis virus. Cell. 1975;5(1):51–8. doi: 10.1016/0092-8674(75)90091-4 . 165900

[7] Abraham G, Banerjee AK. Sequential transcription of the genes of vesicular stomatitis virus. Proc Natl Acad Sci U S A. 1976;73(5):1504–8. Epub 1976/05/01. doi: 10.1073/pnas.73.5.1504 ; PubMed Central PMCID: PMC430325. 179088 PMC430325

[8] Ball LA, White CN. Order of transcription of genes of vesicular stomatitis virus. Proc Natl Acad Sci U S A. 1976;73(2):442–6. Epub 1976/02/01. doi: 10.1073/pnas.73.2.442 ; PubMed Central PMCID: PMC335925. 174107 PMC335925

[9] Stillman EA, Whitt MA. Transcript initiation and 5’-end modifications are separable events during vesicular stomatitis virus transcription. J Virol. 1999;73(9):7199–209. doi: 10.1128/JVI.73.9.7199-7209.1999 . 10438807 PMC104244

[10] Barr JN, Whelan SP, Wertz GW. cis-Acting signals involved in termination of vesicular stomatitis virus mRNA synthesis include the conserved AUAC and the U7 signal for polyadenylation. J Virol. 1997;71(11):8718–25. doi: 10.1128/JVI.71.11.8718-8725.1997 ; PubMed Central PMCID: PMC192336. 9343230 PMC192336

[11] Iverson LE, Rose JK. Localized attenuation and discontinuous synthesis during vesicular stomatitis virus transcription. Cell. 1981;23(2):477–84. Epub 1981/02/01. doi: 10.1016/0092-8674(81)90143-4 . 6258804

[12] Blumberg BM, Leppert M, Kolakofsky D. Interaction of VSV leader RNA and nucleocapsid protein may control VSV genome replication. Cell. 1981;23(3):837–45. Epub 1981/03/01. doi: 10.1016/0092-8674(81)90448-7 . 6261959

[13] Peluso RW, Moyer SA. Viral proteins required for the in vitro replication of vesicular stomatitis virus defective interfering particle genome RNA. Virology. 1988;162(2):369–76. Epub 1988/02/01. doi: 10.1016/0042-6822(88)90477-1 . 2829424

[14] La Ferla FM, Peluso RW. The 1:1 N-NS protein complex of vesicular stomatitis virus is essential for efficient genome replication. J Virol. 1989;63(9):3852–7. Epub 1989/09/01. doi: 10.1128/JVI.63.9.3852-3857.1989 ; PubMed Central PMCID: PMC250979. 2548001 PMC250979

[15] Gupta AK, Banerjee AK. Expression and purification of vesicular stomatitis virus N-P complex from Escherichia coli: role in genome RNA transcription and replication in vitro. J Virol. 1997;71(6):4264–71. Epub 1997/06/01. doi: 10.1128/JVI.71.6.4264-4271.1997 ; PubMed Central PMCID: PMC191641. 9151813 PMC191641

[16] Ogino T, Banerjee AK. Unconventional mechanism of mRNA capping by the RNA-dependent RNA polymerase of vesicular stomatitis virus. Mol Cell. 2007;25(1):85–97. Epub 2007/01/16. S1097-2765(06)00785-4 [pii] doi: 10.1016/j.molcel.2006.11.013 . 17218273

[17] Ogino T, Yadav SP, Banerjee AK. Histidine-mediated RNA transfer to GDP for unique mRNA capping by vesicular stomatitis virus RNA polymerase. Proc Natl Acad Sci U S A. 2010;107(8):3463–8. Epub 2010/02/10. 0913083107 [pii] doi: 10.1073/pnas.0913083107 ; PubMed Central PMCID: PMC2840475. 20142503 PMC2840475

[18] Ogino T, Banerjee AK. The HR motif in the RNA-dependent RNA polymerase L protein of Chandipura virus is required for unconventional mRNA-capping activity. J Gen Virol. 2010;91(Pt 5):1311–4. Epub 2010/01/29. doi: 10.1099/vir.0.019307-0 [pii] ; PubMed Central PMCID: PMC2855774. 20107017 PMC2855774

[19] Ogino M, Ito N, Sugiyama M, Ogino T. The rabies virus L protein catalyzes mRNA capping with GDP polyribonucleotidyltransferase activity. Viruses. 2016;8(5):144. doi: 10.3390/v8050144 ; PubMed Central PMCID: PMC4885099. 27213429 PMC4885099

[20] Ogino T, Banerjee AK. Formation of guanosine(5’)tetraphospho(5’)adenosine cap structure by an unconventional mRNA capping enzyme of vesicular stomatitis virus. J Virol. 2008;82(15):7729–34. Epub 2008/05/23. doi: 10.1128/JVI.00326-08 ; PubMed Central PMCID: PMC2493324. 18495767 PMC2493324

[21] Neubauer J, Ogino M, Green TJ, Ogino T. Signature motifs of GDP polyribonucleotidyltransferase, a non-segmented negative strand RNA viral mRNA capping enzyme, domain in the L protein are required for covalent enzyme-pRNA intermediate formation. Nucleic Acids Res. 2016;44(1):330–41. doi: 10.1093/nar/gkv1286 ; PubMed Central PMCID: PMC4705655. 26602696 PMC4705655

[22] Liang B, Li Z, Jenni S, Rahmeh AA, Morin BM, Grant T, et al. Structure of the L protein of vesicular stomatitis virus from electron cryomicroscopy. Cell. 2015;162(2):314–27. doi: 10.1016/j.cell.2015.06.018 ; PubMed Central PMCID: PMC4557768. 26144317 PMC4557768

[23] Ogino T. Capping of vesicular stomatitis virus pre-mRNA is required for accurate selection of transcription stop-start sites and virus propagation. Nucleic Acids Res. 2014;42(19):12112–25. doi: 10.1093/nar/gku901 ; PubMed Central PMCID: PMC4231761. 25274740 PMC4231761

[24] Ogino M, Ogino T. 5’-Phospho-RNA acceptor specificity of GDP polyribonucleotidyltransferase of vesicular stomatitis virus in mRNA capping. J Virol. 2017;91(6):e02322–16. doi: 10.1128/JVI.02322-16 ; PubMed Central PMCID: PMC5331801. 28053102 PMC5331801

[25] Braun MR, Deflube LR, Noton SL, Mawhorter ME, Tremaglio CZ, Fearns R. RNA elongation by respiratory syncytial virus polymerase is calibrated by conserved region V. PLoS Pathog. 2017;13(12):e1006803. Epub 2017/12/28. doi: 10.1371/journal.ppat.1006803 ; PubMed Central PMCID: PMC5760109. 29281742 PMC5760109

[26] Butcher SJ, Grimes JM, Makeyev EV, Bamford DH, Stuart DI. A mechanism for initiating RNA-dependent RNA polymerization. Nature. 2001;410(6825):235–40. Epub 2001/03/10. doi: 10.1038/35065653 . 11242087

[27] Appleby TC, Perry JK, Murakami E, Barauskas O, Feng J, Cho A, et al. Viral replication. Structural basis for RNA replication by the hepatitis C virus polymerase. Science. 2015;347(6223):771–5. Epub 2015/02/14. doi: 10.1126/science.1259210 . 25678663

[28] Te Velthuis AJ, Robb NC, Kapanidis AN, Fodor E. The role of the priming loop in influenza A virus RNA synthesis. Nat Microbiol. 2016;1:16029. Epub 2016/08/31. doi: 10.1038/nmicrobiol.2016.29 . 27572643

[29] Ogino M, Gupta N, Green TJ, Ogino T. A dual-functional priming-capping loop of rhabdoviral RNA polymerases directs terminal de novo initiation and capping intermediate formation. Nucleic Acids Res. 2019;47(1):299–309. Epub 2018/11/06. doi: 10.1093/nar/gky1058 . 30395342 PMC6326812

[30] Horwitz JA, Jenni S, Harrison SC, Whelan SPJ. Structure of a rabies virus polymerase complex from electron cryo-microscopy. Proc Natl Acad Sci U S A. 2020;117(4):2099–107. Epub 2020/01/19. doi: 10.1073/pnas.1918809117 ; PubMed Central PMCID: PMC6995008. 31953264 PMC6995008

[31] Gilman MSA, Liu C, Fung A, Behera I, Jordan P, Rigaux P, et al. Structure of the Respiratory Syncytial Virus Polymerase Complex. Cell. 2019;179(1):193–204 e14. Epub 2019/09/10. doi: 10.1016/j.cell.2019.08.014 . 31495574 PMC7111336

[32] Pan J, Qian X, Lattmann S, El Sahili A, Yeo TH, Jia H, et al. Structure of the human metapneumovirus polymerase phosphoprotein complex. Nature. 2020;577(7789):275–9. Epub 2019/11/08. doi: 10.1038/s41586-019-1759-1 ; PubMed Central PMCID: PMC6949429. 31698413 PMC6949429

[33] Abdella R, Aggarwal M, Okura T, Lamb RA, He Y. Structure of a paramyxovirus polymerase complex reveals a unique methyltransferase-CTD conformation. Proc Natl Acad Sci U S A. 2020;117(9):4931–41. Epub 2020/02/23. doi: 10.1073/pnas.1919837117 ; PubMed Central PMCID: PMC7060699. 32075920 PMC7060699

[34] Ogino T. In vitro capping and transcription of rhabdoviruses. Methods. 2013;59(2):188–98. doi: 10.1016/j.ymeth.2012.05.013 ; PubMed Central PMCID: PMC3449051. 22687619 PMC3449051

[35] Ogino M, Fedorov Y, Adams DJ, Okada K, Ito N, Sugiyama M, et al. Vesiculopolins, a New Class of Anti-Vesiculoviral Compounds, Inhibit Transcription Initiation of Vesiculoviruses. Viruses. 2019;11(9). Epub 2019/09/22. doi: 10.3390/v11090856 ; PubMed Central PMCID: PMC6783830. 31540123 PMC6783830

[36] Lawson ND, Stillman EA, Whitt MA, Rose JK. Recombinant vesicular stomatitis viruses from DNA. Proc Natl Acad Sci U S A. 1995;92(10):4477–81. doi: 10.1073/pnas.92.10.4477 ; PubMed Central PMCID: PMC41967. 7753828 PMC41967

[37] Jenni S, Bloyet LM, Diaz-Avalos R, Liang B, Whelan SPJ, Grigorieff N, et al. Structure of the Vesicular Stomatitis Virus L Protein in Complex with Its Phosphoprotein Cofactor. Cell Rep. 2020;30(1):53–60 e5. Epub 2020/01/09. doi: 10.1016/j.celrep.2019.12.024 ; PubMed Central PMCID: PMC7049099. 31914397 PMC7049099

[38] Jenni S, Salgado EN, Herrmann T, Li Z, Grant T, Grigorieff N, et al. In situ Structure of Rotavirus VP1 RNA-Dependent RNA Polymerase. J Mol Biol. 2019;431(17):3124–38. Epub 2019/06/25. doi: 10.1016/j.jmb.2019.06.016 ; PubMed Central PMCID: PMC6697194. 31233764 PMC6697194

[39] Juhasz K, Murphy BR, Collins PL. The major attenuating mutations of the respiratory syncytial virus vaccine candidate cpts530/1009 specify temperature-sensitive defects in transcription and replication and a non-temperature-sensitive alteration in mRNA termination. J Virol. 1999;73(6):5176–80. Epub 1999/05/11. doi: 10.1128/JVI.73.6.5176-5180.1999 ; PubMed Central PMCID: PMC112566. 10233984 PMC112566

[40] Cartee TL, Megaw AG, Oomens AG, Wertz GW. Identification of a single amino acid change in the human respiratory syncytial virus L protein that affects transcriptional termination. J Virol. 2003;77(13):7352–60. Epub 2003/06/14. doi: 10.1128/jvi.77.13.7352-7360.2003 ; PubMed Central PMCID: PMC164798. 12805433 PMC164798

[41] Schnell MJ, Buonocore L, Whitt MA, Rose JK. The minimal conserved transcription stop-start signal promotes stable expression of a foreign gene in vesicular stomatitis virus. J Virol. 1996;70(4):2318–23. PubMed Central PMCID: PMC190073. doi: 10.1128/JVI.70.4.2318-2323.1996 8642658 PMC190073

[42] Brown T, Mackey K, Du T. Analysis of RNA by northern and slot blot hybridization. Curr Protoc Mol Biol. 2004;Chapter 4:Unit 4 9. Epub 2008/02/12. doi: 10.1002/0471142727.mb0409s67 18265351

[43] Schneider CA, Rasband WS, Eliceiri KW. NIH Image to ImageJ: 25 years of image analysis. Nat Methods. 2012;9(7):671–5. Epub 2012/08/30. doi: 10.1038/nmeth.2089 ; PubMed Central PMCID: PMC5554542. 22930834 PMC5554542

[44] Crooks GE, Hon G, Chandonia JM, Brenner SE. WebLogo: a sequence logo generator. Genome Res. 2004;14(6):1188–90. doi: 10.1101/gr.849004 ; PubMed Central PMCID: PMC419797. 15173120 PMC419797

[45] Berman HM, Westbrook J, Feng Z, Gilliland G, Bhat TN, Weissig H, et al. The Protein Data Bank. Nucleic Acids Res. 2000;28(1):235–42. Epub 1999/12/11. doi: 10.1093/nar/28.1.235 ; PubMed Central PMCID: PMC102472. 10592235 PMC102472

[46] Waterhouse A, Bertoni M, Bienert S, Studer G, Tauriello G, Gumienny R, et al. SWISS-MODEL: homology modelling of protein structures and complexes. Nucleic Acids Res. 2018;46(W1):W296–W303. Epub 2018/05/23. doi: 10.1093/nar/gky427 ; PubMed Central PMCID: PMC6030848. 29788355 PMC6030848

[47] Krieger E, Joo K, Lee J, Lee J, Raman S, Thompson J, et al. Improving physical realism, stereochemistry, and side-chain accuracy in homology modeling: Four approaches that performed well in CASP8. Proteins. 2009;77 Suppl 9:114–22. Epub 2009/09/22. doi: 10.1002/prot.22570 ; PubMed Central PMCID: PMC2922016. 19768677 PMC2922016

[48] Emsley P, Lohkamp B, Scott WG, Cowtan K. Features and development of Coot. Acta Crystallogr D Biol Crystallogr. 2010;66(Pt 4):486–501. Epub 2010/04/13. doi: 10.1107/S0907444910007493 ; PubMed Central PMCID: PMC2852313. 20383002 PMC2852313

[49] DeLano WL. The PyMOL Molecular Graphics System. http://www.pymol.org/. 2002.

