# Supplementary figures and images for: GDP polyribonucleotidyltransferase domain of vesicular stomatitis virus polymerase regulates leader-promoter escape and polyadenylation-coupled termination during stop-start transcription
Source: PLoS Pathog. 2022 Feb 2;18(2):e1010287. doi: 10.1371/journal.ppat.1010287 (PMC8843114; doi:10.1371/journal.ppat.1010287)

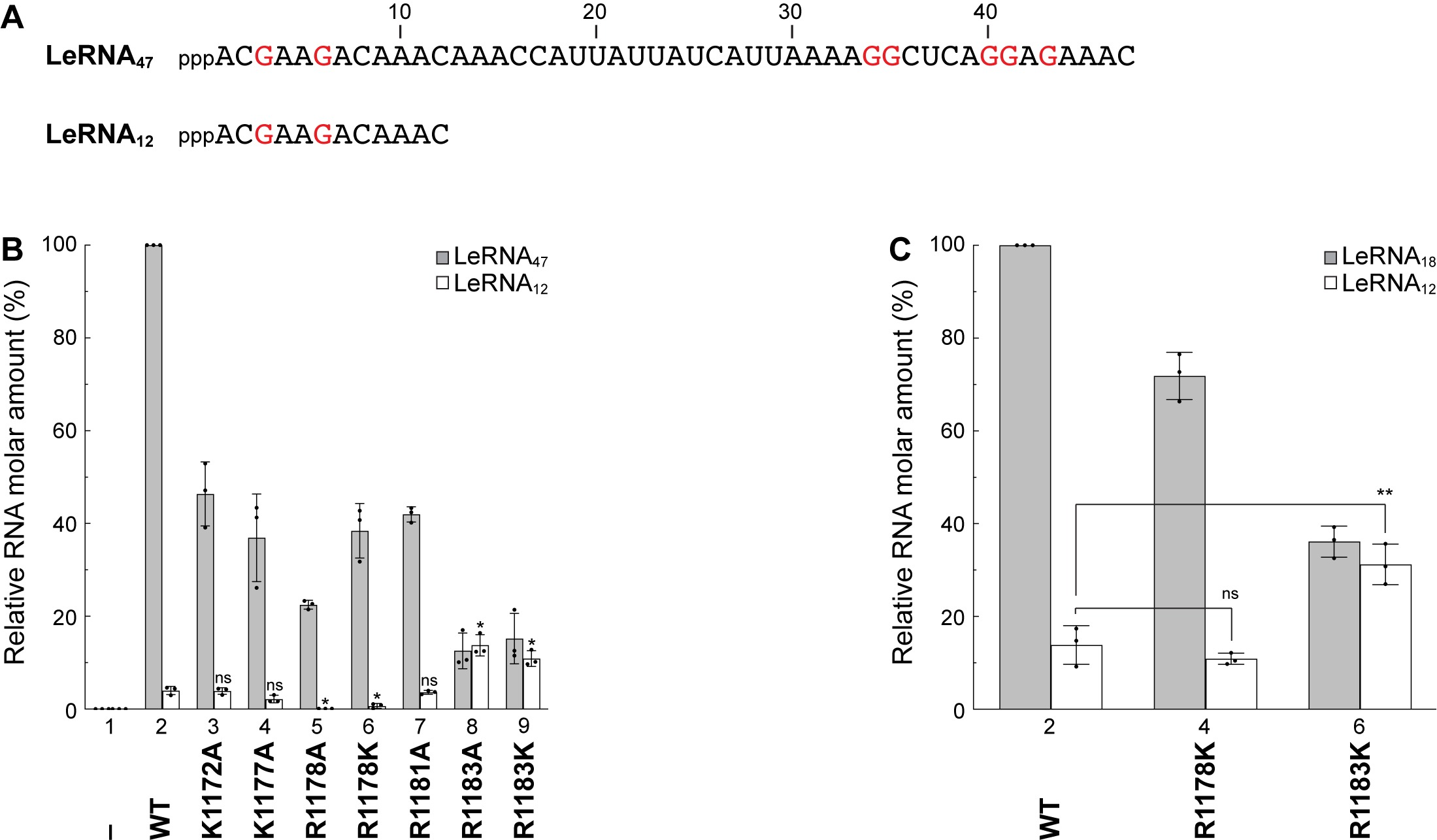

Supplement: S1 Fig — (A) The full-length LeRNA (LeRNA47) and prematurely-terminated LeRNA with ~12 nt (LeRNA12) contain 7 and 2 G residues, respectively, at the indicated positions. (B) Based on the radioactivities of [α-32P]GMP-labeled LeRNA47 and LeRNA12 (Fig 3C, lanes 1–9) and the number of the G residues in these RNAs, relative molar amounts of LeRNA47 (gray columns, the same as in Fig 3C) and LeRNA12 (open columns) synthesized by the WT or mutant L protein during 2-h transcription were estimated. The amount of LeRNA47 synthesized by the WT L protein (Fig 3C, lane 2) was set to 100%. Statistical significance for differences in the amounts of LeRNA12 synthesis by the mutant L proteins compared to the WT L protein (open column 2) was examined by one-way ANOVA [ns, not significant (p ≥ 0.05); *, p < 0.05; **, p <0.01]. (C) Relative molar amounts of LeRNA18 (gray columns) and LeRNA12 (open columns) synthesized by the WT or mutant L protein during 3-min transcription in the absence of UTP (Fig 4B, lanes 2, 4, and 6) were estimated. The amount of LeRNA18 synthesized by the WT L protein (Fig 4B, lane 2) was set to 100%. Statistical significance for differences in the amounts of LeRNA12 synthesis by the mutant L proteins compared to the WT L protein (open column 2) was examined by one-way ANOVA. (TIF) [file ppat.1010287.s001.tif]

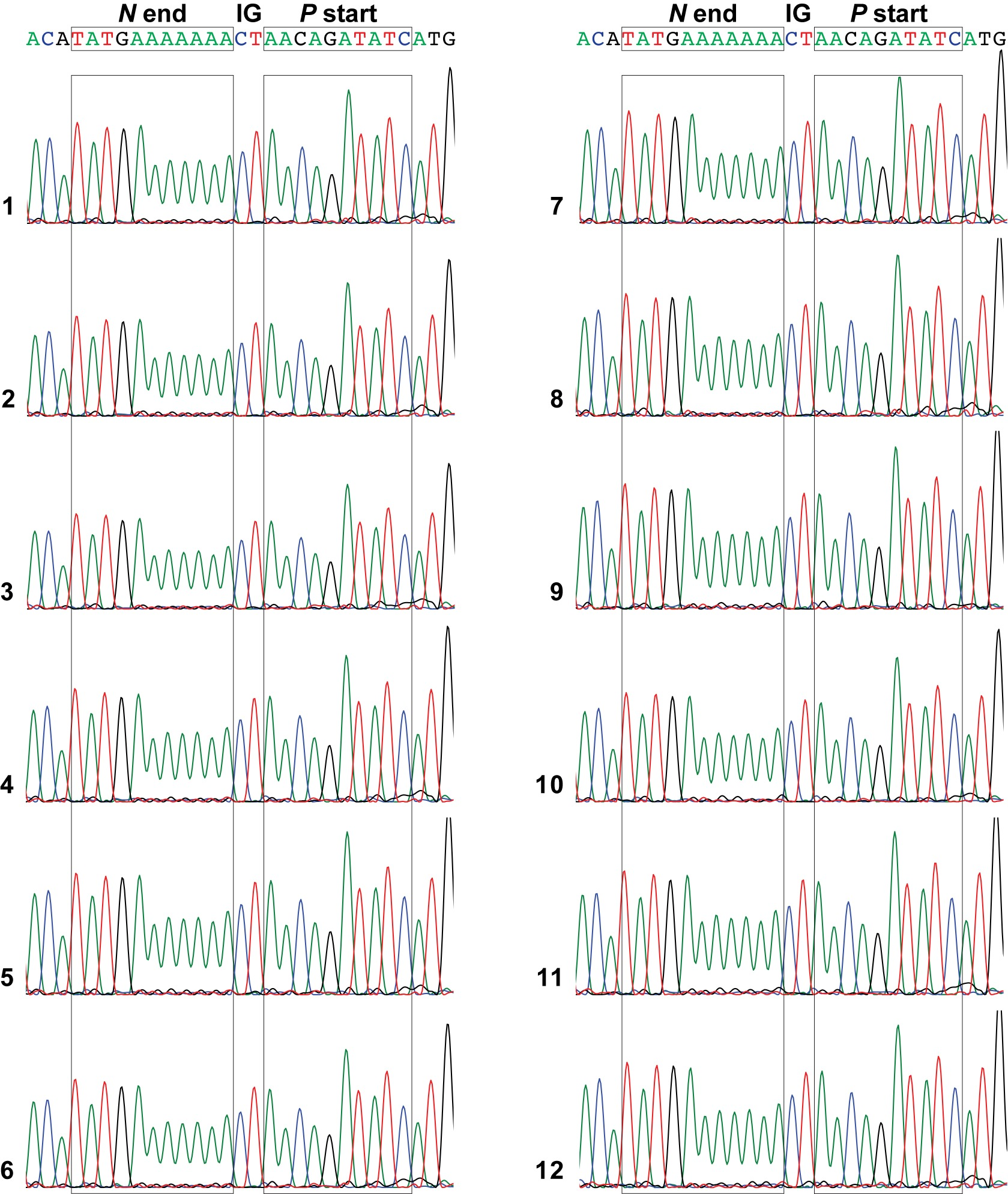

Supplement: S2 Fig — The N-P junction in 12 cDNA clones derived from polyadenylated polycistronic mRNAs synthesized by the R1178K mutant L protein in vitro were sequenced. Electropherograms are shown with the N-P junction sequence (top). The N gene-end and P gene-start sequences are boxed. IG indicates the intergenic sequence. (TIF) [file ppat.1010287.s002.tif]

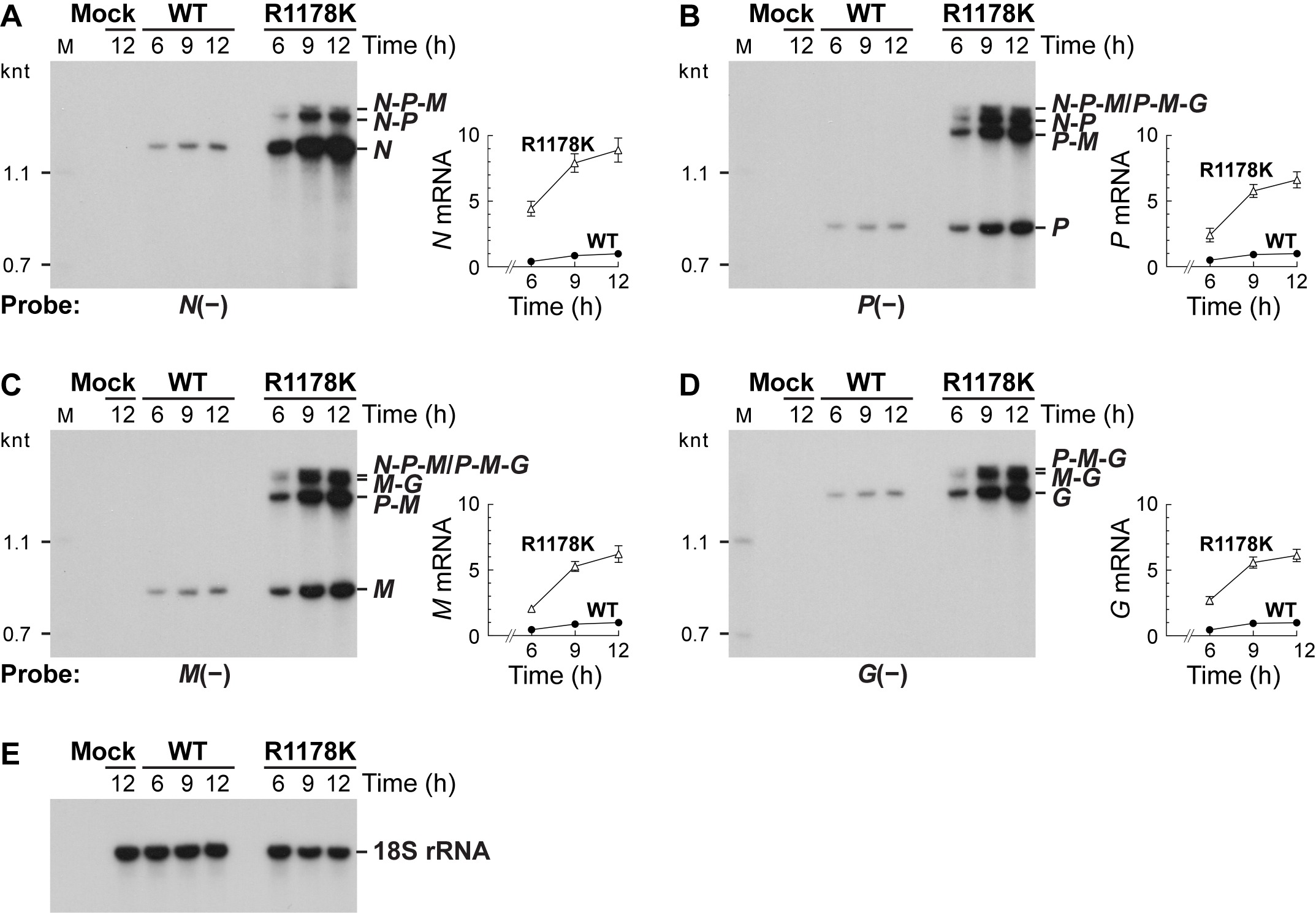

Supplement: S3 Fig — BHK-21 cells were mock-infected or infected with the WT or R1178K mutant rVSV at a multiplicity of infection of 5 and cultured at 37°C. Total RNAs were extracted from the cells at 6-h, 9-h, and 12-h post-infection and treated with RNase H in the presence of oligo(dT). The RNase H-treated RNAs (0.1 μg) were analyzed by Northern blotting sequentially with the probes against N (A), P (B), M (C), and G (D) mRNAs as in Fig 5. The graphs show relative amounts of monocistronic mRNAs synthesized in cells infected with the WT (closed circles) or R1178K mutant (open triangles) rVSV. The amounts of the viral monocistronic mRNAs in the total RNAs from the WT virus-infected cells at 12-h post-infection was set to 1. Symbols and error bars represent the means and standard deviations, respectively, of three independent experiments (n = 3). (E) Cellular 18S rRNA on the same membrane was detected with an antisense probe. (TIF) [file ppat.1010287.s003.tif]

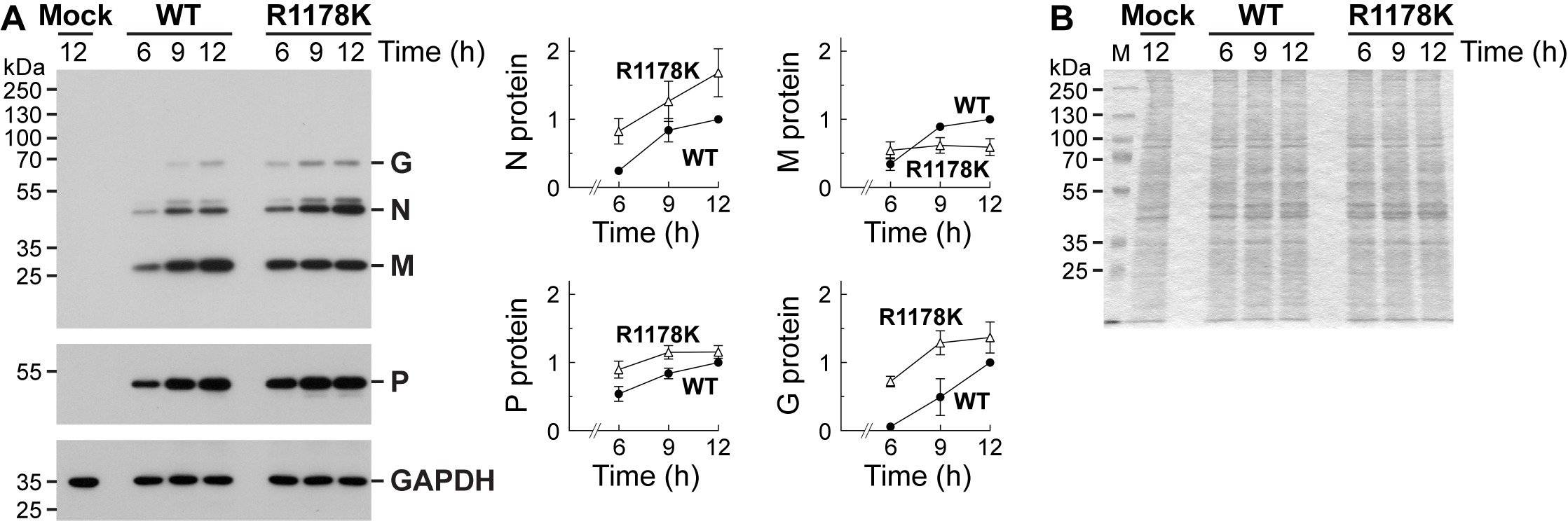

Supplement: S4 Fig — BHK-21 cells were mock-infected or infected with the WT or R1178K mutant rVSV as in S3 Fig. Cell lysates were prepared at 6-h, 9-h, and 12-h post-infection. (A) The cell lysates (1 μg protein) were analyzed by Western blotting with rabbit anti-VSV (N, M, and G proteins), -P, or -GAPDH polyclonal antibody. The graphs show relative amounts of the N, P, M, and G proteins synthesized in cells infected with the WT (closed circles) or R1178K mutant (open triangles) rVSV. The amounts of the viral proteins in the lysates from the WT virus-infected cells at 12-h post-infection was set to 1. Symbols and error bars represent the means and standard deviations, respectively, of three independent experiments (n = 3). (B) The cell lysates (10 μg protein) were analyzed by 10% SDS-PAGE followed by staining with Coomassie Brilliant Blue. (TIF) [file ppat.1010287.s004.tif]

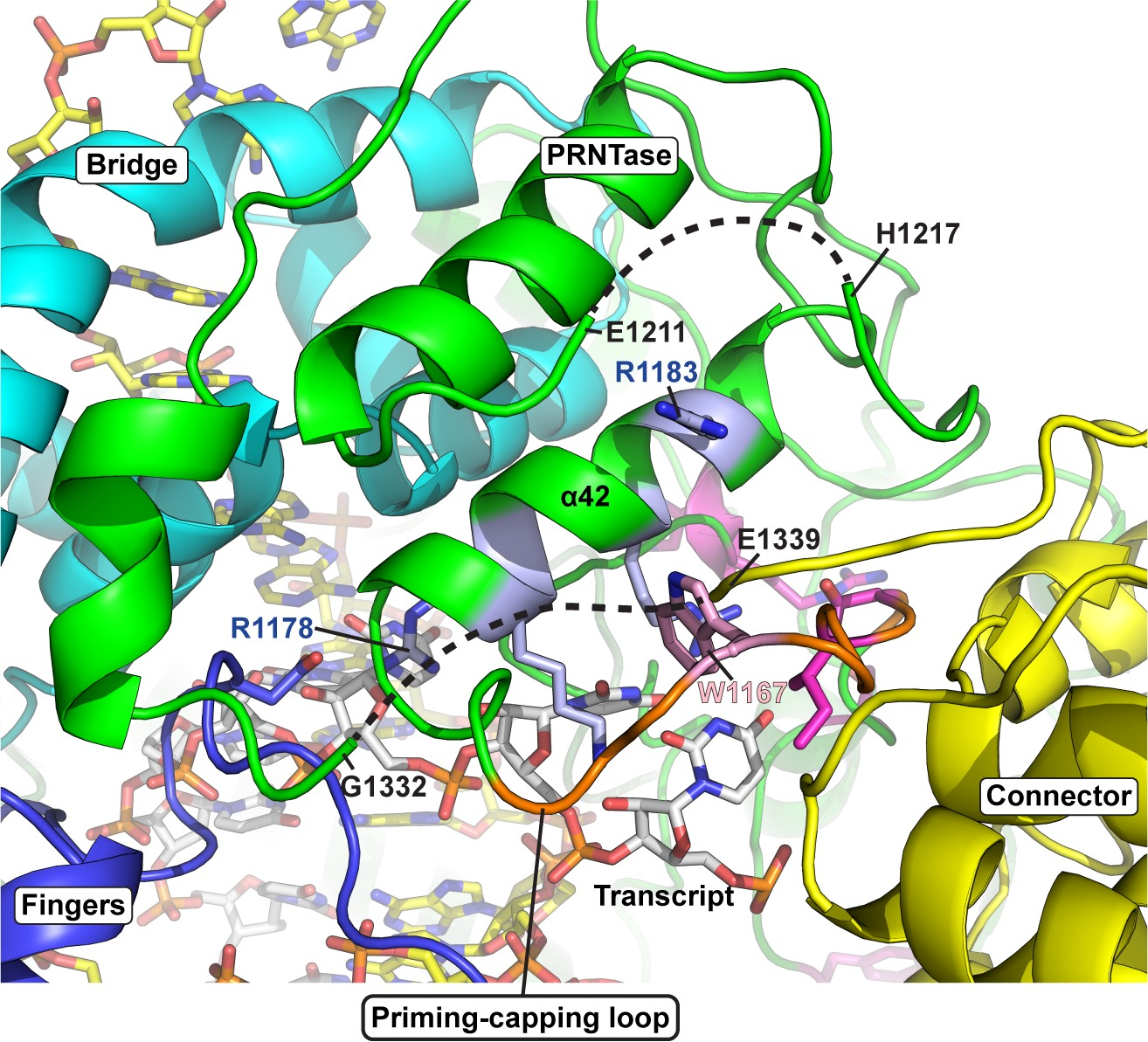

Supplement: S5 Fig — The model of a putative elongation complex of the VSV L protein is shown with colors as in Fig 7. The connector domain (yellow) is placed based on superposition of the modeled polymerase core and with the intact VSV polymerase model (PDB id: 6U1X). R1183 sits on the opposite face of helix α42 by comparison to R1178. The side chain of R1183 faces both a loop within the PRNTase and the linker between the PRNTase and connector domain. Residues between 1211–7 (on the PRNTase loop) and 1332–9 (in the linker) are not resolved in PDB id: 6U1X and are connected by a dashed-line. (TIF) [file ppat.1010287.s005.tif]

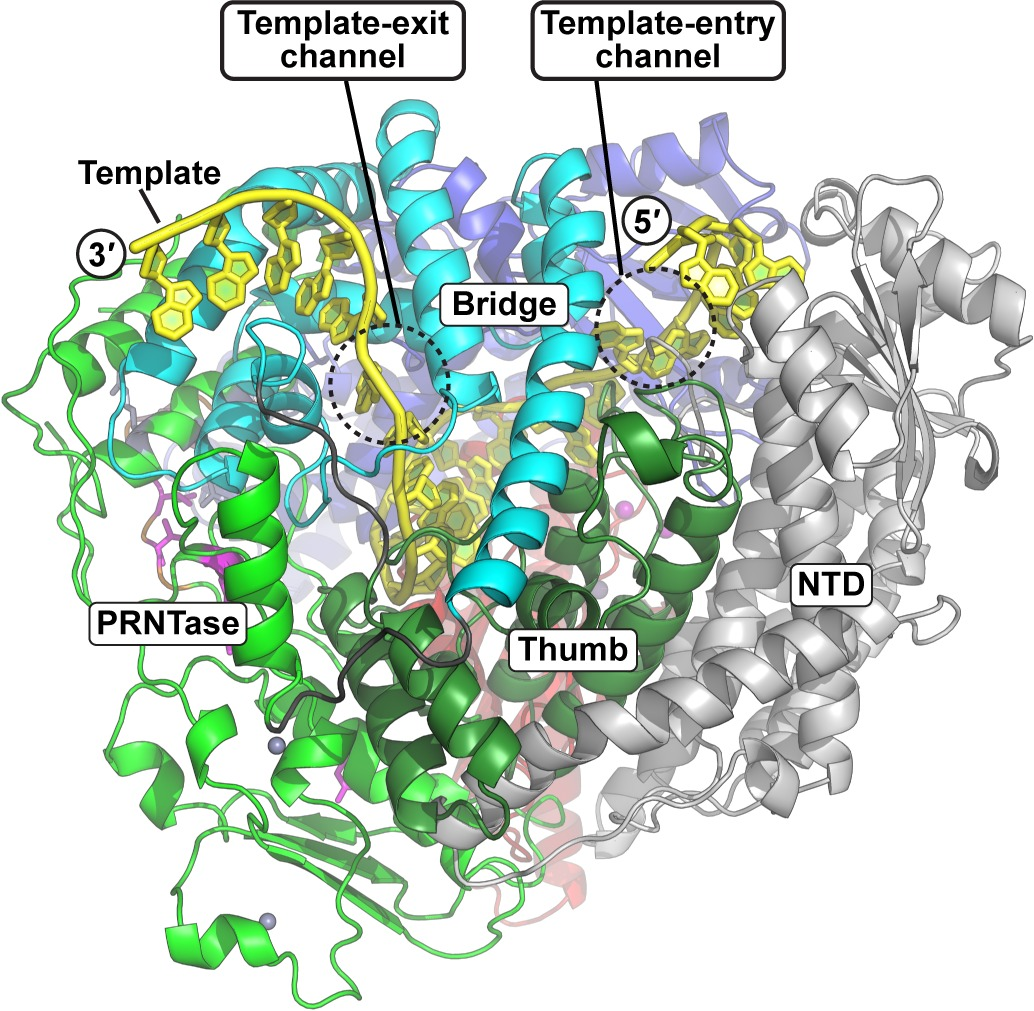

Supplement: S6 Fig — The modeled structure of the VSV L elongation complex shown in Fig 7A is viewed from a different angle. (TIF) [file ppat.1010287.s006.tif]

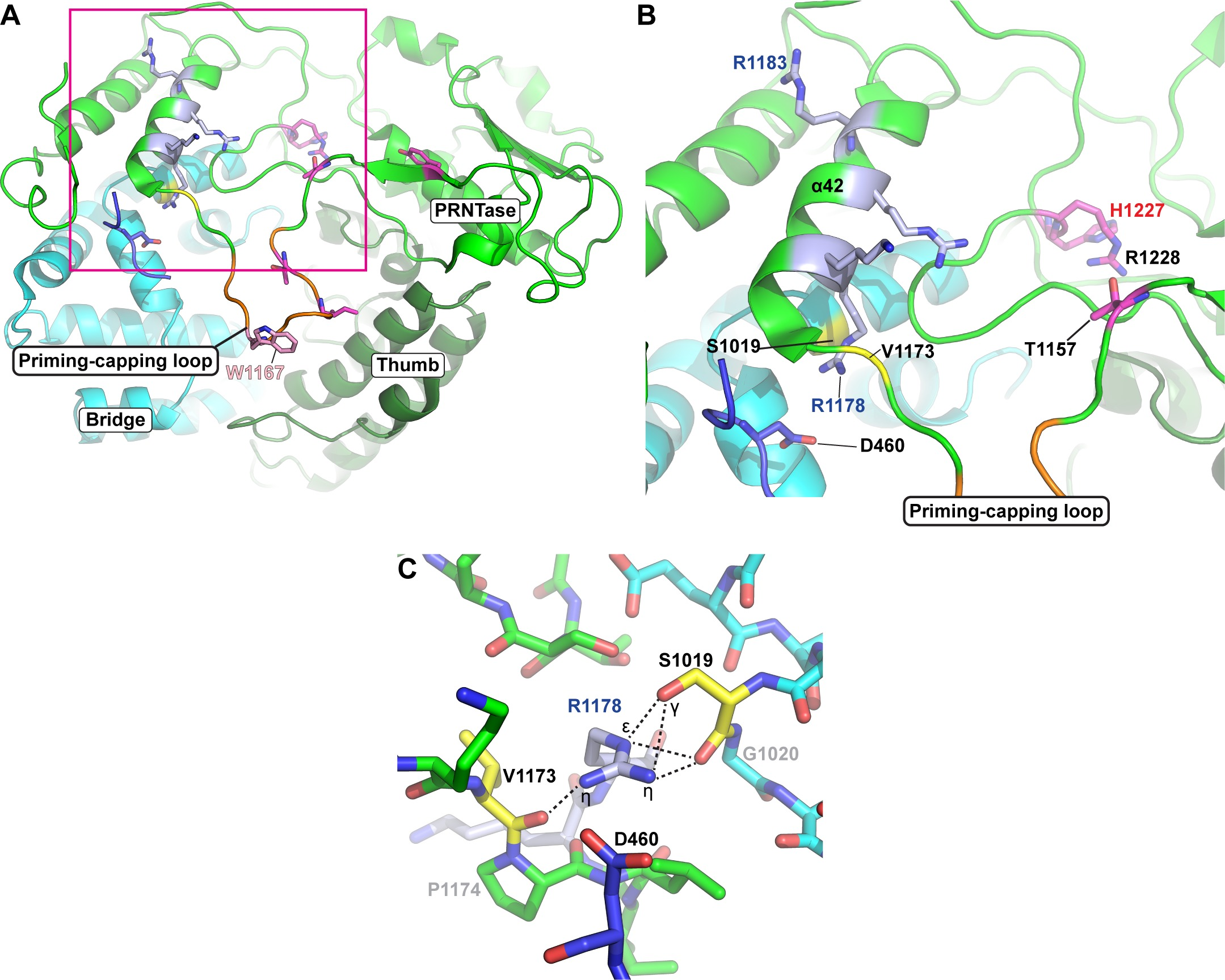

Supplement: S7 Fig — In (A), a panned out view of the L protein in absence of RNA, noted as the apo-state in the text, is shown in the context of R1178. Secondary structure elements and key residues (shown as sticks) surrounding R1178 are shown with colors as in Fig 7, though residues S1019 and V1173 are shaded yellow here. (B) shows a close-up view of the direct environment of R1178, with local and key functional residues noted. (C) shows an alternate stick model for a subset of residues in the vicinity of R1178. Residue interactions with guanidinium group of the R1178 side chain are noted with dashed lines. Carbon-backbone colors, in (C), correspond to colors as in (A) and (B). Models in this figure were generated from coordinates in PDB id: 6U1X. For clarity, the N-terminal, fingers (excluding residues 457–62), palm, and thumb subdomain are not shown. (TIF) [file ppat.1010287.s007.tif]
